# Probing of coupling effect induced plasmonic charge accumulation for water oxidation

**DOI:** 10.1093/nsr/nwaa151

**Published:** 2020-07-06

**Authors:** Yuying Gao, Feng Cheng, Weina Fang, Xiaoguo Liu, Shengyang Wang, Wei Nie, Ruotian Chen, Sheng Ye, Jian Zhu, Hongyu An, Chunhai Fan, Fengtao Fan, Can Li

**Affiliations:** State Key Laboratory of Catalysis, Dalian National Laboratory for Clean Energy, Collaborative Innovation Center of Chemistry for Energy Materials (iChEM), Dalian Institute of Chemical Physics, Chinese Academy of Sciences, Dalian 116023, China; University of Chinese Academy of Sciences, Beijing 100049, China; State Key Laboratory of Catalysis, Dalian National Laboratory for Clean Energy, Collaborative Innovation Center of Chemistry for Energy Materials (iChEM), Dalian Institute of Chemical Physics, Chinese Academy of Sciences, Dalian 116023, China; School of Chemistry and Chemical Engineering, and Institute of Molecular Medicine, Renji Hospital, School of Medicine, Shanghai Jiao Tong University, Shanghai 200240, China; School of Chemistry and Chemical Engineering, and Institute of Molecular Medicine, Renji Hospital, School of Medicine, Shanghai Jiao Tong University, Shanghai 200240, China; State Key Laboratory of Catalysis, Dalian National Laboratory for Clean Energy, Collaborative Innovation Center of Chemistry for Energy Materials (iChEM), Dalian Institute of Chemical Physics, Chinese Academy of Sciences, Dalian 116023, China; State Key Laboratory of Catalysis, Dalian National Laboratory for Clean Energy, Collaborative Innovation Center of Chemistry for Energy Materials (iChEM), Dalian Institute of Chemical Physics, Chinese Academy of Sciences, Dalian 116023, China; University of Chinese Academy of Sciences, Beijing 100049, China; State Key Laboratory of Catalysis, Dalian National Laboratory for Clean Energy, Collaborative Innovation Center of Chemistry for Energy Materials (iChEM), Dalian Institute of Chemical Physics, Chinese Academy of Sciences, Dalian 116023, China; State Key Laboratory of Catalysis, Dalian National Laboratory for Clean Energy, Collaborative Innovation Center of Chemistry for Energy Materials (iChEM), Dalian Institute of Chemical Physics, Chinese Academy of Sciences, Dalian 116023, China; State Key Laboratory of Catalysis, Dalian National Laboratory for Clean Energy, Collaborative Innovation Center of Chemistry for Energy Materials (iChEM), Dalian Institute of Chemical Physics, Chinese Academy of Sciences, Dalian 116023, China; State Key Laboratory of Catalysis, Dalian National Laboratory for Clean Energy, Collaborative Innovation Center of Chemistry for Energy Materials (iChEM), Dalian Institute of Chemical Physics, Chinese Academy of Sciences, Dalian 116023, China; School of Chemistry and Chemical Engineering, and Institute of Molecular Medicine, Renji Hospital, School of Medicine, Shanghai Jiao Tong University, Shanghai 200240, China; State Key Laboratory of Catalysis, Dalian National Laboratory for Clean Energy, Collaborative Innovation Center of Chemistry for Energy Materials (iChEM), Dalian Institute of Chemical Physics, Chinese Academy of Sciences, Dalian 116023, China; State Key Laboratory of Catalysis, Dalian National Laboratory for Clean Energy, Collaborative Innovation Center of Chemistry for Energy Materials (iChEM), Dalian Institute of Chemical Physics, Chinese Academy of Sciences, Dalian 116023, China

**Keywords:** plasmonic photocatalysis, coupling effect, charge separation, surface photovoltage, spatial distribution

## Abstract

A key issue for redox reactions in plasmon-induced photocatalysis, particularly for water oxidation, is the concentration of surface-accumulating charges (electrons or holes) at a reaction site for artificial photosynthesis. However, where plasmonic charge accumulated at a catalyst's surface, and how to improve local charge density at active sites, remains unknown because it is difficult to identify the exact spatial location and local density of the plasmon-induced charge, particularly with regard to holes. Herein, we show that at the single particle level, plasmon-coupling-induced holes can be greatly accumulated at the plasmonic Au nanoparticle dimer/TiO_2_ interface in the nanogap region, as directly evidenced by the locally enhanced surface photovoltage. Such an accumulation of plasmonic holes can significantly accelerate the water oxidation reaction (multi-holes involved) at the interfacial reaction site, with nearly one order of magnitude enhancement in photocatalytic activities compared to those of highly dispersed Au nanoparticles on TiO_2_. Combining Kelvin probe force microscopy and theoretical simulation, we further clarified that the local accumulated hole density is proportional to the square of the local near-field enhancement. Our findings advance the understanding of how charges spatially distribute in plasmonic systems and the specific role that local charge density at reaction sites plays in plasmonic photocatalysis.

## Teaser

Plasmon coupled nanostructure severing as light-harvesting centers was demonstrated to generate a high density of holes at catalytic sites, which significantly accelerate photocatalytic water oxidation reaction.

## INTRODUCTION

Plasmonic metal nanoparticles (NPs) have broadly tunable optical properties, providing impressive applications in many areas, including solar energy conversion [1–6] and surface-enhanced spectroscopy [7,8]. Plasmonic NPs serving as optical antennas can highly amplify the local optical field on the surface, enabling the generation of a high concentration of hot charges, electrons and holes [9]. These charges have demonstrated potential in visible-light-induced photochemical reactions [3,10,11], in particular in water oxidation (2H_2_O→O_2_+4H^+^+4e^−^) [12,13], which is regarded as the primary reaction for natural and artificial photosynthesis but is the bottleneck of artificial photosynthetic systems because of its sluggish reaction kinetics and charge dynamics involving multi-electron/proton transfer processes [14–16]. This suggests that the local hole density at oxygen evolution sites must be sufficiently high for the water oxidation reaction [17].

In natural photosynthesis, efficient light harvesting and fast charge transfer to reaction center can be achieved through the construction of light-harvesting antennas containing hundreds of functional pigments surrounding a reaction center of photosystem I and II, acting as a local funnel [18], while in artificial photosynthesis, only a few energetic charges survive because of the severe charge recombination during their diffusion transfer to the reaction sites. This situation makes it even more challenging for plasmonic photocatalysts to accumulate high density holes at reaction sites during the short time for H_2_O oxidation because of the extremely short lifetime of plasmonic holes (femtosecond to picosecond). Although integrating plasmonic NPs with a semiconductor can generate a Schottky barrier at their interface to achieve charge spatial separation prolonging the charge lifetime [4,5,19–21], it is still difficult to extract a sufficient high holes concentration for water oxidation reaction [13].

Plasmonic charges (electrons and holes) generated from surface plasmon resonance (SPR) decay in a few femtoseconds through a nonradiative channel [22]. Recently, considerable theoretical research efforts have been undertaken to explore the effects of geometry, particle size and electronic structure on plasmon decay channels to optimize the charge generation [23,24]. One effective mean of increasing the plasmonic charge generation efficiency is to design dimers that may act as an optical receiving antenna [25,26], concentrating the incident light into their nanogap. Integrating metal nanoparticle dimers (NDs) into semiconductors has demonstrated the possibility of increasing the quantum efficiency of charge separation [25]. However, the spatial accumulation site of the separated charge and the determinants of local charge density in a metal ND/semiconductor are poorly understood. Here, we show experimentally that a high density of spatially separated holes can be confined to the Au NDs/TiO_2_ interface in the nanogap region induced by a strong local electromagnetic field sustained by plasmon coupling between Au NDs. By combining experimental results and theoretical calculations, we demonstrate that the local hole density is proportional to the square of the near-field enhancement intensity. Compared with Au NPs, the high confined hole density in Au NDs/TiO_2_ exhibits nearly one order of magnitude enhancement in the photocatalytic activity, indicating the importance of plasmon coupling effect and charge density in water oxidation.

## RESULTS AND DISCUSSION

**Figure 1.**
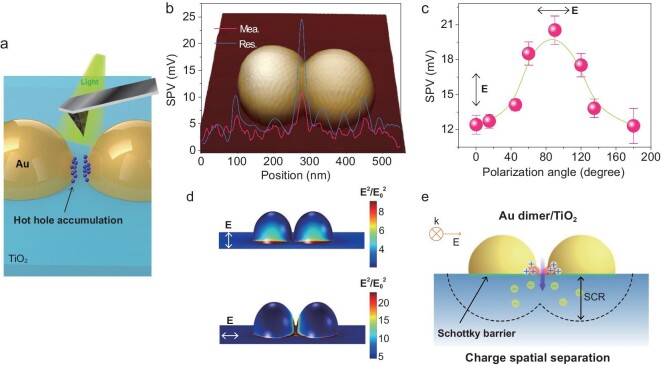
Plasmon-induced charge spatial distribution of Au dimer/TiO_2_ nanostructure. (a) Schematic of Kelvin Probe Force Microscopy measurement of the Au nanoparticle dimers (NDs) on the TiO_2_ substrate. Plasmonic holes confined at Au/TiO_2_ interface which locates nanogap region between Au NDs. (b) Measured (Mea.) and restored (Res.) SPV profiles along the center horizontal line of the Au NDs under 620 nm light illumination. Inset: three-dimensional (3D) atomic force microscopy (AFM) image of Au NDs/TiO_2_. (c) SPV measured at the spot of Au NDs/TiO_2_ interface as a function of incident light polarization angle. The black bidirectional arrows indicate the direction of the light polarization. The angle 0° corresponds to the polarization perpendicular to the Au NDs axis. Error bars indicate the statistical uncertainty in the SPV. (d) Sectional view of the simulated electromagnetic (EM) field intensity distribution when the polarization angles are 0° and 90°. (e) Schematic illustration of plasmon-induced charge spatial separation of the Au NDs/TiO_2_ system. SCR stands for surface charge region. The downward purple arrow means the direction of plasmonic electron transfer from Au NPs to TiO_2_.

Figure 1a shows the experimental scheme for probing charge spatial distribution in Au NDs/TiO_2_ plasmonic photocatalyst using Kelvin Probe Force Microscopy (KPFM), which measures spatial variation in surface potential across a sample surface [27–30]. With a combination of excitation light sources, KPFM images the surface potential change (i.e. surface photovoltage, SPV) distribution [28], which is directly correlated to the spatially separated charge distribution. Figure 1b shows that Au NDs were assembled on TiO_2_ by moving two nearly identical Au NPs using atomic force microscopy (AFM) nanomanipulation. This method can control the interparticle distance of Au NDs at a nanometer accuracy [31]. The separation distance of Au NDs is 7 nm. The size of the Au NPs is 64 nm (Supplementary Fig. 1). The Au NPs on the TiO_2_ substrate in the shape of hemispheres form a well-defined metal–oxide interface (Supplementary Figs 2 and 3). Conductive-AFM measurement shows a Schottky barrier of 0.82 eV at Au/TiO_2_ interface (Supplementary Fig. 4), which not only allows the plasmonic electron transfer into TiO_2_ but also hinders the back flow of electrons from TiO_2_ to Au. Under 620 nm light excitation, the SPV profile along the dimer axis shows the spatial heterogeneity of charge distribution (Fig. 1b). Notably, the SPV at the Au dimer/TiO_2_ interface in the nanogap region is considerably larger than that of the side spots at the outer ends of the Au dimer. Such an SPR-induced SPV signal is not observed for the Au@SiO_2_/TiO_2_ system in which the Au/TiO_2_ interface was modified by a thin SiO_2_ layer (Supplementary Fig. 5), suggesting that direct interfacial contact at Au/TiO_2_ is required for plasmon-induced charge spatial separation. Besides, the contact between Au and TiO_2_ generated hybrid states can stabilize plasmonic holes from Au NPs [13]. Because SPV reflects the local accumulation or depletion of photogenerated charges on the surface and is directly proportional to the local charge density upon light excitation (see details in Supplementary Fig. 6) [28,32], its sign is also sensitive to the direction of charge transfer, i.e. the type of photogenerated majority charge accumulated on the surface [33]. To rule out the possible contribution from plasmonic photothermal effects (details in Supplementary data), the light intensity used in the KPFM experiments was maintained at a moderate level (0.5 mW/cm^2^) in KPFM experiments guaranteeing Au dimer's stabilization. We observed signatures of a positive SPV signal at Au NPs/TiO_2_ interface under plasmon excitation, which is attributed to charge spatial separation (Supplementary Fig. 7). We also demonstrated that the Au 4f_7/2_ of Au/TiO_2_ heterostructures shifted to the higher binding energies under SPR excitation compared to that in the dark, strongly suggesting that plasmonic electrons were transferred from Au to TiO_2_ (Supplementary Fig. 3). While a single SPV peak at Au NDs/TiO_2_ interface in the nanogap region is present due to the limitation of spatial resolution (Fig. 1b), the enhanced positive SPV is a direct indication that a high density of holes are confined to their interface in the nanogap region.

To further quantify the SPV results, namely, the high density of holes at the Au dimer/TiO_2_ interface, we carefully considered the cross-talk and tip convolution effect. Control experiments were carried out by considering the geometric effect of the tip and sample and evaluating the effect of the tip–sample distance in the lift mode on the measured surface potential and SPV (Supplementary Figs 8–11). It was found that the SPV signal is less affected by the cross-talk effect. To minimize the unavoidable finite-size tip convolution effect, an efficient algorithm was used to restore the actual SPV [34]. The restored SPV signal at the Au NDs/TiO_2_ interface in the nanogap region is noticeably larger than anywhere else in the sample (Fig. 1b). We further calculated surface accumulating charge concentration on plasmonic nanostructure, and the unit of local density of spatially separated holes is nm^−2^. The results show that the density of spatially separated holes at the coupled Au NDs/TiO_2_ interface can be up to 4 × 10^−3^ nm^−2^ (details in Supplementary data), which is 6–7 times higher than that of highly dispersed Au NPs/TiO_2_ (6 × 10^−4^ nm^−2^).

Figure 1c shows the plasmon-induced SPV under different polarization. The observed SPV shows a trigonometric function shape at angles from 0° to 180°, with a maximum value at 90°. To determine the origin of this observation, we simulated the electromagnetic field of this system for polarizations along and orthogonal to the interparticle axis (Fig. 1d). For an incident polarization perpendicular to the interparticle axis (0°), a fully uncoupled state of Au NDs/TiO_2_ produces a weak near-field intensity at their interface in the nanogap region [35,36]. In contrast, the light polarization is parallel to the Au NDs interparticle axis (90°), the cophasing of the electron oscillation in the Au NDs creates a near-field amplified by a factor of 183, known as near-field ‘hot spots’ in this system, leading to an enhanced SPV signal. The role of Au NDs in the local charge density recognized here is reminiscent of the pigment role in natural photosynthesis. This is why a high density of holes in a coupled Au NDs/TiO_2_ acts as an optical antenna, enhancing the optical field intensity at the Au NDs/TiO_2_ interface as well as the charge generation [26] and leads to the accumulation of a high density of spatially separated holes at the Au NDs/TiO_2_ interface (Fig. 1e).

**Figure 2.**
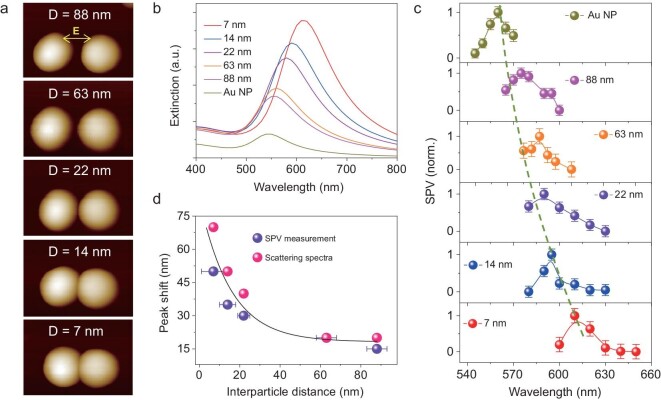
Surface photovoltage (SPV) measurement and calculated extinction spectra of Au NP/TiO_2_ and Au NDs/TiO_2_ with different interparticle distances. (a) AFM images of Au NDs/TiO_2_ with varying separation distance (D) on a TiO_2_ substrate. (b) Calculated extinction spectra of Au NP/TiO_2_ and Au NDs/TiO_2_. (c) SPV spectra comparison of Au NP/TiO_2_ and Au NDs/TiO_2_ structures. In these measurements, the polarization is parallel to the Au dimer axis. The green dashed line is the maximum SPV as a guide to the eye for the red-shift of the SPV peak. The normalized SPV signals are shown for easy comparison. Error bars in the plots indicate the statistical uncertainty in the SPV. (d) SPV and SPR peak shift versus the separation distance of the Au dimers compared to Au NP. The black curve is fitted by single-exponential function.

To elucidate how the coupling intensity affects the charge density distribution, an Au NDs/TiO_2_ with different separation distances was investigated. Figure 2a shows AFM topographic images of the Au NDs/TiO_2_ with five different distances (Ds) of 88, 63, 22, 14 and 7 nm. The separation distances were determined using a model calculation to render the tip–sample convolution effect (Supplementary Fig. 12) [37]. A pronounced red shift of the SPR peak is observed with decreasing separation distance (Fig. 2b), which can be interpreted using a dipole–dipole model [38]. Under SPR excitation, SPV distribution was primarily located at the nanogap (Supplementary Fig. 13). SPV at the Au NDs/TiO_2_ interface was measured as a function of light wavelength (Fig. 2c). With decreasing gap distance, the wavelengths of the SPV maxima show bathochromic shifts, as indicated by the green dashed line in Fig. 2c. To consider the effect of electron transfer on plasmon resonance wavelength, a two-electrode system equipped with dark-field microscopy was used to measure single-particle scattering spectroscopy at different applied bias voltages (Supplementary Fig. 14). We found that the interfacial electron transfer does not affect the plasmon resonance wavelength. The SPV spectra of the Au NDs/TiO_2_ were found to faithfully correspond with the scattering spectra, as clearly proven by the comparison of peak positions of the SPV and scattering shown in Fig. 2d. The SPR wavelength exponentially decays with the interparticle distance, coinciding with the universal scaling behavior of coupled plasmonic nanostructures [39]. The dependence of SPV on separation distance and illumination wavelength is a signature of the plasmonic dimer nanostructure and strong evidence of the plasmon coupling between the Au NDs.

By illuminating the Au NDs/TiO_2_ at their resonant wavelengths with a polarization of 90°, nearly all Au NDs/TiO_2_ with different interparticle distances showed enhanced SPV signals at their interface in the nanogap region (Supplementary Fig. 15). These results are similar to those shown in Fig. 1b but with different SPV intensities by varying the interparticle distance. In contrast, under a polarization of 0°, the intensity of the SPV signal at the Au NDs/TiO_2_ interface in the nanogap region has nearly no enhancement compared to that at the outer-side spots (Supplementary Fig. 16). These results demonstrated that the density of the spatially separated holes at the Au NDs/TiO_2_ interface is dependent on both the light polarization and gap distance, suggesting that near-field coupling enhancement determines local hole density.

**Figure 3.**
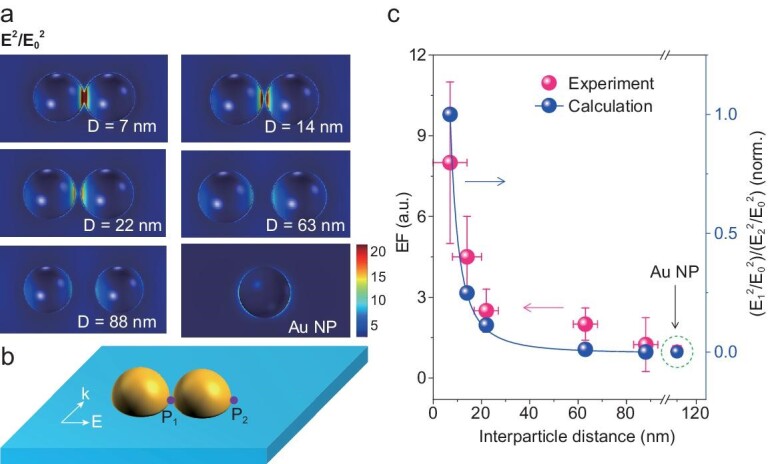
Spatial distribution of the calculated near-field enhancement and the experimental SPV EF of Au NP/TiO_2_ and Au dimers/TiO_2_ with varying gap distance (D). (a) Spatial distribution of the calculated near-field intensity enhancement (defined as $| {{{\rm{E}}^2}/{\rm{E}}_0^{{\rm{\ }}2}} |$) at plasmon resonance excitation. (b) Schematic of the definition of P_1_ and P_2_. P_1_ corresponds to the Au NDs/TiO_2_ interface in the nanogap region, and P_2_ corresponds to the region of the Au NDs/TiO_2_ extremities. (c) SPV EF and the square of the calculated near-field intensity enhancement of Au NP/TiO_2_ and Au NDs/TiO_2_ as a function of the dimer interparticle distance. E_1_ and E_2_ correspond to the electric field intensity at P_1_ and P_2_, respectively. Error bars in the plots indicate the statistical uncertainty of the SPV.

To quantitatively describe the correlation between the charge density and the plasmonic near-field intensity of the Au NDs/TiO_2_ (${{\rm{E}}_{{\rm{ND}}}}$), we calculated the square of the near-field intensity enhancement ($| {{{\rm{E}}_{{\rm{ND}}}}^2/{\rm{E}}_0^{{\rm{\ }}2}} |$, where ${{\rm{E}}_0}$ is incident electric field intensity) distribution of the Au NDs/TiO_2_ nanostructure under horizontal polarized light irradiation (Fig. 3a). Plasmonic near-field coupling intensity at the nanogap was significantly enhanced with decreasing interparticle distance. To intuitively evaluate the enhancement in the density of spatially separated holes, we defined an SPV enhancement factor (EF) as the ratio between the SPV magnitudes detected at Au NDs/TiO_2_ interface inside the nanogap (P_1_) and outside of the dimer (P_2_), as shown in Fig. 3b. The SPV EF shows an inverse dependence on the interparticle distance (Fig. 3c). The result is reminiscent of that of surface-enhanced Raman spectroscopy, which indicates that the Raman signal enhancement factor can be modified by changing the interparticle distance of the Au dimer [40]. To evaluate the correlation between the SPV and the local near-field intensity, the ratio of the near-field enhancement at P_1_ to that at P_2_ for Au NDs with different distances was calculated according to the line profiles of the near-field distribution (Supplementary Fig. 17). Interestingly, the decay trend of the SPV EF versus interparticle distance coincides with that of the square of the enhanced near-field intensity, indicating that the density of the spatially separated holes is proportional to the square of the amplitude of the enhanced near-field. This qualitative relationship is supported by the quantum theory in metal NPs [41,42]. Considering a monochromatic plane–wave interaction with the Au NDs/TiO_2_ nanostructure, the optical field intensity at the sample surface can be written as follows:

(1) \begin{equation*}I\!\!\left(r \right) = {\rm{\ }}p\!\left(r \right)\frac{{{c_0}\sqrt {{\varepsilon _0}} }}{{2\pi }}{E_0}^2,\end{equation*}

where *r* is arbitrary spatial location on Au ND/TiO_2_ surface, *p*(*r*) is the amplification magnitude of the local optical field at an arbitrary position in space, ${\varepsilon _0}$ is the optical dielectric constant of air, ${c_0}$ is a constant and ${E_0}$ is the incident electric field intensity. From the theory based on the equation of motion of the density matrix and the Fowler law for electron injection probability [41], the number of injected electrons or remaining holes is obtained as follows:

(2) \begin{equation*}{\rm{Q}}\!\!\left( r \right)\! \propto\! \frac{{{{\left| {p\!\left( r \right)\!{E_0}} \right|}^2}}}{{\hbar {\pi ^2}}}{\left( {\hbar {\rm{\omega }} - {\varphi _B}} \right)^2},\end{equation*}

where ${\rm{Q}}\!(r)$ describes the amount of spatially separated charge resulting from plasmon-induced interfacial charge transfer, which is directly correlated with the SPV signal. Thus, the SPV signal can be conveniently expressed in terms of the electric field as follows:

(3) \begin{equation*}{\rm{SPV}}\!\left( r \right){\rm{\ }} \propto {\rm{\ }}{\left| {p\!\left(r \right)\!{E_0}} \right|^2},\end{equation*}

which is consistent with the experimental observation (Fig. 3c). According to the aforementioned consideration, plasmonic optical antenna with multiple hot spots can create a high density of photogenerated charges, which is the prerequisite for multi-charge involved photocatalytic oxidation reactions, in particular, for the water oxidation reaction.

**Figure 4.**
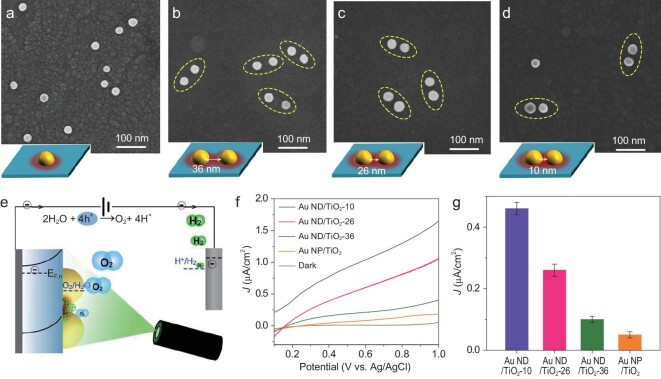
Architecture and photoelectrochemical performance of plasmonic photoanodes. (a–d) SEM images of Au NP/TiO_2_ and Au NDs/TiO_2_ photoanodes. The insets show the schematics of the plasmonic samples. (e) Schematic of the photoelectrochemical measurement of an Au NDs/TiO_2_ electrode. (f) Current versus potential characteristics of plasmonic photoanodes recorded in a 1 M aqueous Na_2_SO_4_ solution under visible-light irradiation (>420 nm). (g) Photocurrent density of Au NP/TiO_2_ and Au NDs/TiO_2_ photoelectrodes at a fixed applied potential of 0.5 V versus Ag/AgCl.

Among the most challenging and important photocatalytic reactions, water oxidation is regarded as the primary reaction for natural and artificial photosynthesis. Water oxidation involves successive oxidation of intermediates, which needs to accumulate sufficient holes at reaction sites to effectively drive the multi-hole transfer processes [16]. Such a reaction is particularly suitable to verify the critical role played by the surface-accumulating hole in a catalytic reaction. Plasmonic holes have been demonstrated to be capable of achieving water oxidation at the Au NPs/TiO_2_ interface under visible light [13]. Theoretical simulations indicated that the rate-determining step for oxygen evolution is the generation of ^*^OOH intermediate with an activation energy barrier of ∼3.0 eV [13]. The highly oxidized intermediate requires the next transport of hole from reaction center to generate oxygen molecule within a short time. The local charge density at reaction sites thus plays a critical role for efficient water oxidation. Here, Au NDs/TiO_2_ samples possess notable superiority in tuning hole density at hot spots by varying the interparticle distance. Thus, we used visible-light-driven water oxidation as a probe reaction to elucidate the effect of hole density at hot spots on the reaction. The scanning electron microscopy (SEM) image in Fig. 4a shows Au NPs dispersed on TiO_2_ film, but the interparticle distance exceeds 100 nm, suggesting the absence of plasmon-coupling effect in this sample. To verify whether plasmon coupling of Au NDs is favorable for reaction activity enhancement, Au NDs/TiO_2_ samples were prepared using DNA super-origami as templates (see details in Supplementary data; Supplementary Fig. 18). Figure 4b and c show the SEM images of the Au NDs/TiO_2_ sample with a separation distance of 36 nm (signed as Au ND/TiO_2_-36) and 26 nm (signed as Au ND/TiO_2_-26), which is within the scope of the plasmon coupling but with a weak coupling according to Fig. 3c. When the Au ND separation distance was further decreased to 10 nm (signed as Au ND/TiO_2_-10) (Fig. 4d), the plasmon coupling strength greatly increased. Since the local density of accumulating plasmonic holes at hot spots of coupled Au ND/TiO_2_ photocatalyst is lower than the generally recognized inflection point (1 nm^−2^) from first- to third-order reaction mechanism for water oxidation [43], we consider the rate-determining step for water oxidation remains unchanged under coupling effect. It is thus anticipated that more plasmonic holes will accumulate at the Au NDs/TiO_2_ interface for effectively triggering the water oxidation reaction.

Photoelectrochemical (PEC) water oxidation performance of Au NDs/TiO_2_ photoanodes under visible-light irradiation (λ > 420 nm) were examined in a three-electrode cell with Pt foil as the counter electrode and Ag/AgCl as the reference electrode (Fig. 4e). The surface coverage of Au nanoparticles for all samples used in photoelectrochemical experiments is 2%. As displayed in Fig. 4f, Au NDs/TiO_2_ samples significantly outperform Au NPs/TiO_2_ in PEC water oxidation under potentials from 0.1 to 1.0 V_Ag/AgCl_. As the separation distance of Au dimers decreased, photocurrent density is dramatically increased due to the strong plasmon coupling. The negative shift of onset potential of the Au ND/TiO_2_-10 sample can be attributed to the extreme high density of plasmonic holes accumulated at the reaction site. Chopped photocurrent–time curves show that the steady photocurrents of the Au NDs/TiO_2_-10 is up to 0.46 μA/cm^2^ at 0.5 V_Ag/AgCl_, which is higher than that of Au NPs/TiO_2_ (0.05 μA/cm^2^) (Supplementary Fig. 19a). It should be noted that the photocurrent density of the Au NDs/TiO_2_ sample for water oxidation is low, which is attributed to low surface coverage of Au dimers. Moreover, there is no photocurrent observed for the Au-free/TiO_2_ sample, suggesting that the enhanced photocatalytic activity was induced by plasmonic effect. To verify the production of O_2_ molecules, the concentration of dissolved oxygen was monitored with fluorescence-based O_2_ sensor for Au NDs/TiO_2_-10 over one hour (Supplementary Fig. 19b). The concentration is increased with prolonging photocatalytic reaction time. Figure 4g provides the quantitative correlation between the water oxidation activity and the Au ND separation distance. Au NPs/TiO_2_ can also initiate water oxidation reaction and show the least activity. For Au NDs/TiO_2_ electrode, the water oxidation activity is greatly enhanced and its activity is further enhanced when the separation distance is shortened from 36 to 10 nm. Photocurrent density of Au NDs/TiO_2_-10 for plasmon-induced water oxidation is about one order of magnitude higher compared with the Au NP/TiO_2_ photoanode, following the relationship between SPV EF and separation distance recognized in the experiment (Fig. 3c).

Photocurrent shows the dependence on excitation wavelength and the action spectrum qualitatively tracks the SPR absorption spectrum of the Au ND/TiO_2_ sample (Supplementary Fig. 20a), which convincingly demonstrates that the surface plasmonic effect is responsible for the observed water oxidation activity. Electrochemical impedance spectroscopy (EIS) measurements show a smaller arc radius of Au ND/TiO_2_-10 compared to the Au NP/TiO_2_ sample (Supplementary Fig. 21a and Table S1), demonstrating that plasmon coupling effect can also facilitate the charge transfer process from Au ND/TiO_2_ to redox species. Open circuit photovoltage (OCP) analysis further demonstrates that plasmon coupling of Au NDs plays a critical role in the density of accumulated charges for water oxidation (Supplementary Fig. 21b). The observed linear dependence of catalytic activities and the SPV on the light intensity for Au/TiO_2_ also indicates that the high density of holes accumulated at the reaction site is favorable for the water oxidation reaction (Supplementary Figs 6 and 21). These results show that multiple holes accumulated at plasmonic photocatalyst reaction sites play a crucial role during water oxidation. Au ND/TiO_2_ exhibited good stability within 300 s, and photocurrent decreased with increasing time due to the amplification of separation distance of Au dimers (Supplementary Fig. 22).

## CONCLUSION

In conclusion, we demonstrated a plasmonic coupling effect in Au NDs/TiO_2_ that provides intense near-field at its interface in the nanogap region, enabling a high density of spatially separated holes accumulated at hot spots, as proven by the enhanced SPV signal. We showed that it is possible to tune the local charge density by varying the separation distance, light polarization and illumination wavelength. By combining these results with theoretical simulation, we further determined the quantitative relationship between the local charge density and near-field enhancement. We further explored the effect of the local charge accumulation of the Au NDs/TiO_2_ on the multi-hole driven water oxidation reaction and observed nearly one order of magnitude enhancement in the catalytic activity compared to that of the Au NP/TiO_2_ sample. Thus, as well as identifying the spatial sites of charge ‘hot spots’, the results demonstrated that the plasmonic coupling effect in a metal/semiconductor nanostructure generates a previously unrecognized effect on accumulating charge for a multi-electron/hole catalytic reaction. This study provides insight into the extraction and management of charge in a plasmonic photocatalytic system.

## METHODS

### Fabrication of Au NP/TiO_2_ and Au dimers/TiO_2_ nanostructure

Au NPs solution was prepared according to the literature [44]. Au NPs were then deposited on Nb-rutile (100) crystal (0.05 wt% Nb-doped, 10 × 10 × 0.5 mm, Shinkosha) using spin-coating method. Before KPFM measurements, the Au NPs/TiO_2_ was exposed to O_2_ plasma and thermal treatment. A detailed description of this process is available in Supplementary data. Au dimers solution was synthesized using super-origami as templates for anchoring Au NPs through DNA hybridization [45]. For PEC measurements, Au dimers were deposited on TiO_2_ film by adsorption 15 min. TiO_2_ thin films were deposited onto the indium tin oxide substrate using a chamber-type Atomic Layer Deposition reactor (home built). A detailed description of this process is available in Supplementary data.

### Characterization

SEM images were taken using a field-emission scanning electron microscope (FE-SEM, JEOL JSM-7800F) at an operating voltage of 20 kV. High-resolution transmission electron microscopy (HR-TEM) images were obtained on a FEI Tecnai G2 F30 S-Twin (FEI Company) with an accelerating voltage of 300 kV for measuring the Au NP sizes. X-ray photoelectron spectroscopy (XPS) measurements were performed on a VG ESCALAB MK2 spectrometer with monochromatized Al-Kα excitation.

### KPFM measurement

Surface potential of the samples were measured using an amplitude-modulated (AM) KPFM (Bruker Dimension V SPM system) in a dual-scan technique. The recorded signals of topography and surface potential arise from their respective retrace scanning processes, reducing the risk of cross-talk effects between the topography and surface potential channels. Surface photovoltage (SPV) was the difference in surface potential under illumination and in the dark, which directly related to the photogenerated charge distribution with nanometer spatial resolution. The test parameters and detailed description of this process is available in Supplementary data.

### PEC measurement

PEC measurements were performed in a three-electrode glass cell with a quartz window containing an aqueous solution of 1 M Na_2_SO_4_. Pt foil was used as the counter electrode and Ag/AgCl as the reference electrode. A 300-W Xenon lamp was used as the light source. A long-pass filter (>420 nm) was used to obtain visible light for PEC measurement. The fluorescence-based O_2_ sensor (Neofox, Ocean Optics) was used to test the dissolved oxygen in reaction solution. An N_2_-gas was used to purge the O_2_ of reaction solution before illumination.

## Supplementary Material

nwaa151_Supplemental_File
