# Supplementary material for: Probing of coupling effect induced plasmonic charge accumulation for water oxidation
Source: Natl Sci Rev. 2020 Jul 6;8(6):nwaa151. doi: 10.1093/nsr/nwaa151 (PMC8288172; doi:10.1093/nsr/nwaa151)
Supplement: nwaa151_Supplemental_File [file nwaa151_supplemental_file.docx]

Supplementary Information for

**Probing of coupling effect induced plasmonic charge accumulation for water oxidation**

Yuying Gao,^1,2,#^ Feng Cheng,^1,#^ Weina Fang,^3^ Xiaoguo Liu,^3^ Shengyang Wang,^1^ Wei Nie,^1,2^ Ruotian Chen,^1^ Sheng Ye,^1^ Jian Zhu,^1^ Hongyu An,^1^ Chunhai Fan,^3^ Fengtao Fan,^1,*^ and Can Li^1,*^

^1^State Key Laboratory of Catalysis, Dalian National Laboratory for Clean Energy, The Collaborative Innovation Centre of Chemistry for Energy Materials (iChEM), Dalian Institute of Chemical Physics, Chinese Academy of Sciences, Dalian 116023, China

^2^University of Chinese Academy of Sciences, Beijing, 100049, China

^3^School of Chemistry and Chemical Engineering, and Institute of Molecular Medicine, Renji Hospital, School of Medicine, Shanghai Jiao Tong University, Shanghai 200240, China

^#^Equally contributed to this work.

^*^Correspondence: ftfan@dicp.ac.cn; canli@dicp.ac.cn

**Contents:**

**S1. Experiment method**

**S2. Characterization of Au NPs and Au/TiO_2_ samples**

**S3. Schottky barrier height measurement of Au/TiO_2_ using conductive atomic force microscope (C-AFM)**

**S4. KPFM measurements of Au/TiO_2_ nanostructure**

**S5. Geometry deconvolution procedures**

**S6. Evaluation the photothermal effect of Au NDs/TiO_2_**

**S7. KPFM measurements and electromagnetic field simulation of Au dimer/TiO_2_**

**S8. Calculating plasmonicholes density of Au/TiO_2_**

**S9. Plasmonic Au NP/TiO_2_ and Au ND/TiO_2_ photoanode**

Figures 1 to 22

References

**S1. Experiment methods**

**Synthesis of Au nanoparticles (NPs)**

200 mL of chloroauric acid (0.28 M) was added to a round-bottom flask and boiled with stirring. 1.4 mL sodium citrate was then added into the boiling solution, followed by vigorous stirring. The resulting reaction mixture was boiled for 30 min. The Au NPs were separated from the reaction mixture by centrifugation. The resulting solution was placed in a refrigerator at 4°C for later use.

**Synthesis of Au NP/TiO_2_ sample**

Nb-rutile (100) crystal (0.05 wt% Nb-doped, 10 × 10 × 0.5 mm, Shinkosha) was used as a semiconducting substrate. The TiO_2_ substrate was rinsed with acetone, ethanol, and deionized water by sonication for 30 min, followed by drying at 80°C for 30 min in an oven. Next, the substrate was immersed in 10% HF solution for 5 min, thoroughly rinsed with deionized water, and dried. Finally, the rutile crystal was annealed at 900°C for 1 h under atmospheric conditions to obtain a flat and clean surface. Au NPs were then dispersed onto the rutile crystal substrate using spin-coating (1500 rpm) of the Au NP solution for 1 min. The Au/TiO_2_ sample was exposed to O_2_ plasma for 10 min to remove surface sodium citrate absorbed on the Au NP surfaces. The final sample was annealed at 400°C for 2 h, and then cooled to room temperature naturally.

**Synthesis of Au@SiO_2_ core-shell NPs**

30 mL Au NP solution and 0.4 mL (3-aminopropyl) trimethoxysilane (1 mM) were added into a round-bottom flask and stirred for 10 min. 3.2 mL of sodium silicate solution (diluted to 0.54 wt% with deionized water and adjusted to pH ~10.2 with hydrochloric acid) was then added the solution, followed by vigorous stirring for 3 min at 25°C. The resulting solution was placed in a 90°C water bath for 30 min. The Au@SiO_2_ solution was diluted into water and centrifuged two more times to remove the excess reactants. The resulted solution was stored at 4°C for future use.

**Synthesis of Au dimer/TiO_2_ sample**

Au dimer solution was synthesized using super-origami as templates for anchoring 40-nm Au NPs on prescribed docking sites through DNA hybridization. The triangle DNA origami monomers were prepared according to literature [1]. The colloidal solutions of 40 nm Au NPs were functionalized with thiolated DNA according to a previously published method [2]. The DNA-modified Au NP solution was added to a DNA origami solution (purified) in a TAE-Mg^2+^ (24 mM tris, 12 mM acetic acid, 1.2 mM EDTA, and 7.5 mM magnesium acetate (pH 8.0)) buffer with a ratio of 2:1 for the 40 nm Au NP dimer. The mixture was annealed from 15 to 45°C at a rate of 0.1°C/min to promote DNA hybridization on the Au NPs. Successful formation of desired structures and separation from other products was accomplished via gel electrophoresis in a 0.5% agarose gel run at 100 V and maintained at 4°C by a surrounding ice bath. Desired bands were cut out and DNA origami-templated Au NP dimers were extracted from the gel using a protocol method [3]. Titanium dioxide thin films with thickness of 40 nm were deposited onto the indium tin oxide substrate using a chamber-type Atomic Layer Deposition reactor (home built). The films were treated using O_2_ plasma for 2 min. A total of 20 μl of the prepared DNA origami-templated Au NP dimers was deposited onto the TiO_2_ film (1×2 cm^2^) and placed in air for 15 min. Then, the sample was rinsed using 1 ml of deionized water and followed by drying at 60°C for 2 h. Finally, the resultant sample was annealed in air at 400°C for 2 h to remove the DNA origami and form an ideal interface contact between the Au and TiO_2_.

**Synthesis of Ag NPs/TiO_2_ sample**

Ag NPs were prepared using ascorbic acid to reduce AgCl in aqueous solution and at ambient conditions. The obtained solution was preserved in the refrigerator at 4°C. The Ag/TiO_2_ sample was prepared by depositing drops of the Ag NP solution on TiO_2_ crystal, followed by drying the sample on a hotplate at 200°C for 20 min with subsequent annealing for 2 h at 300°C in Ar atmosphere.

**Kelvin probe force microscopy (KPFM) measurement**

Amplitude-modulated (AM) KPFM (Bruker Dimension V SPM system) is employed to measure the topography and surface potential of samples at interest area. The AM-mode KPFM offers a high signal-to-noise ratio compared to the frequency modulation KPFM [4]. In AM-KPFM measurement, an AC voltage (V_AC_, 0.5 V in amplitude and 50 kHz in frequency) plus a DC voltage (V_DC_) were applied to the AFM tip. V_AC_ generated oscillating electrostatic forces between the tip and sample surface and V_DC_ compensated for the oscillating electrostatic forces between the AFM tip and the sample. The tip used was a SCM-PIT (Bruker, coated with Pt/Ir) with f_res_ = 75 kHz, r_radius_ = 25 nm, and k = 3 N/m. The nominal radius of the tip curvature was 20 nm. The AFM and surface potential images were recorded in ambient conditions at a scan rate of 0.5 Hz and a resolution of 256 × 256 pixels. The measured surface potential represents the surface work function difference between the tip and sample. As the long-range electrostatic interaction between the probe and sample was detected using AM KPFM, the measured surface potential signal was caused by an averaging effect between the adjacent nanostructures with different surface potentials. To minimize the averaging effect, we tuned the KPFM system to operate at a smaller tip–sample distance (10 nm) that allowed high spatial resolution and stable surface potential signals. Because the surface potential change (i.e. surface photovoltage) is less affected by the tip–sample distances, the surface potential change was used to determine the local charge density. Surface photovoltage (SPV) was the difference in surface potential under illumination and in the dark. SPV originated from the local photogenerated charge density change, which enabled the direct mapping of the photogenerated charge distribution with nanometer spatial resolution. The same tip was used to record surface potential images before and after Au NP SPR excitation to rule out the effect of possible morphologic changes during scanning processes on measurements. The monochromatic visible-light source was derived from a Xe lamp (500 W, Beijing Perfectlight Co. Ltd) equipped with a double-prism monochromator (Zolix Omni-300) and a 350 nm bandpass filter. The monochromatic light was focused to a sub-millimeter spot using a condenser and then irradiated on the sample under oblique incident conditions. To obtain polarized light, a half-wave plate (Thorlabs LPVIS) was inserted into the optical path before the light reached the sample. A 532 nm continuous-wave laser was used to excite the surface plasmons of a single Au NP in the light intensity control experiment. The different light intensities were acquired by rotating a gradient attenuation filter. A light power meter was used to obtain different intensities of the incident light. The data processing was conducted using Nanoscope Analysis software.

**Conductive-AFM (C-AFM) measurement**

The current−voltage characteristics of the samples were investigated using C-AFM with both conductive Pt/Ir tips (Bruker). A direct current voltage was applied between the tip and the sample. The typical contact force used during scanning was approximately 20 nN because large forces can easily scrape the NPs off the TiO_2_ substrate. To prepare the Au/TiO_2_ for C-AFM measurements, the Ag electrode was prepared with Ag glue for good electrical conductivity. Before the I–V curve measurement, a scanning image was first taken over the targeted region. Then, the conductive probe was positioned at the Au particle surface and the current was measured while the bias voltages changed from −2 to +5 V.

**AFM nanomanipulation of Au dimers on TiO_2_ substrate**

We apply a nanomanipulation (Nano man mode, Bruker Dimension V SPM system) technique to assemble Au NP dimers on the rutile (100) crystal substrate. The SCM-PIT probe was used to laterally push the Au NPs lying on the substrate. Controlling the force applied the probe and the distance between tip and substrate surface through a digital panel enabled us to construct a variety of Au dimers with different separation distances. Successful nanomanipulation also depended on the geometry of the probe tip and the direction of the force vector. When pushing a particle, the probe stopped oscillation and maintained a distance of 10 nm from the substrate surface. After this manipulation process, the sample topography was re-imaged using the conventional KPFM mode.

**Numerical simulations**

Plasmonic near-electromagnetic-field distribution of the Au dimers/TiO_2_ nanostructure was performed in the frequency domain with the commercial finite element solver COMSOL using the Wave Optics package [5]. The polarization of the incident electric field was along the dimer axis. The Au dimer separation distances of the model were consistent with those in the experiment conditions. The polarization and wavelength of the excitation light were set in agreement with the experiment conditions. All the maps were generated outside the Au NPs at a distance of 2 nm above the Au surface, which approximates the sample surface to reflect the real electromagnetic field intensity. The mesh size was 5 nm in the simulations. The whole model was a cube surrounded by a perfectly matched layer. Its lower part was a TiO_2_ substrate with the NP placed in the center of the top side and the rest of the model is air. The permittivity of Au, the TiO_2_ substrate, and the surrounding air were taken as −4.624–2.4125i, 2.6, and 1, respectively [6,7]. Extinction cross-section was calculated by summing the absorption and scattering cross-sections. When the scattering spectra were simulated, the background electric field was taken as a plane wave polarized along the interparticle axis. Its wavelength was swept from 400 nm to 800 nm while the corresponding permittivity of Au was taken from the data given by P. B. Johnson and R. W. Christy [6]. The scattered energy was calculated by integrating the Poynting vector over an imaginary sphere surrounding the NPs: W_sca_=∯P∙ndS. Scattering spectra were formed from the scattered energies at discrete wavelengths at intervals of 10 nm.

**Reconstruction of surface potential**

The effects of tip/cantilever convolution may cause an apparent smoothing and averaging in the measured surface potential because of the finite size of the tip [8]. It is necessary to reconstruct the surface potential and SPV using an efficient algorithm [9]. The measured potential, $V$, is affected by all of the charges on the surface of the sample. The dependence of $V$ on the actual surface potential, $\phi$, can be expressed as:

$V\left( r \right)=\int h\left( \left| r-r^{'} \right| \right)\phi\left( r^{'} \right)dr^{'}$ , (1)

where $r$ is the horizontal projection of the tip on the surface of the sample. The contribution of $\phi\left( r' \right)$ to $V\left( r \right)$is weighted by the point spread function, $h\left( \left| r-r' \right| \right)$, which is affected by the size and shape of the tip and the tip–sample distance during measurement. The systemic point spread function was developed according to the previous report [9]. In discrete form, equation (1) can be expressed as:

$V_{i}=\sum_{j} H_{ij}\Phi_{j}$ , (2)

where $V_{i}$ is the potential measured when the tip is above $r_{i}$, $\Phi_{j}$ is the actual potential at $r_{j}$, and $H_{ij}=h\left( \left| r_{i}-r_{j} \right| \right)$. To restore $\Phi_{j}$, we optimized them by minimizing $\sum_{i} \left( V_{i}-\sum_{j} H_{ij}\Phi_{j} \right)^{2}$. Finally, the actual potentials acquired were smoothed to filter out the effect of noise.

**S2. Characterization of Au NPs and Au/TiO_2_ samples**

**
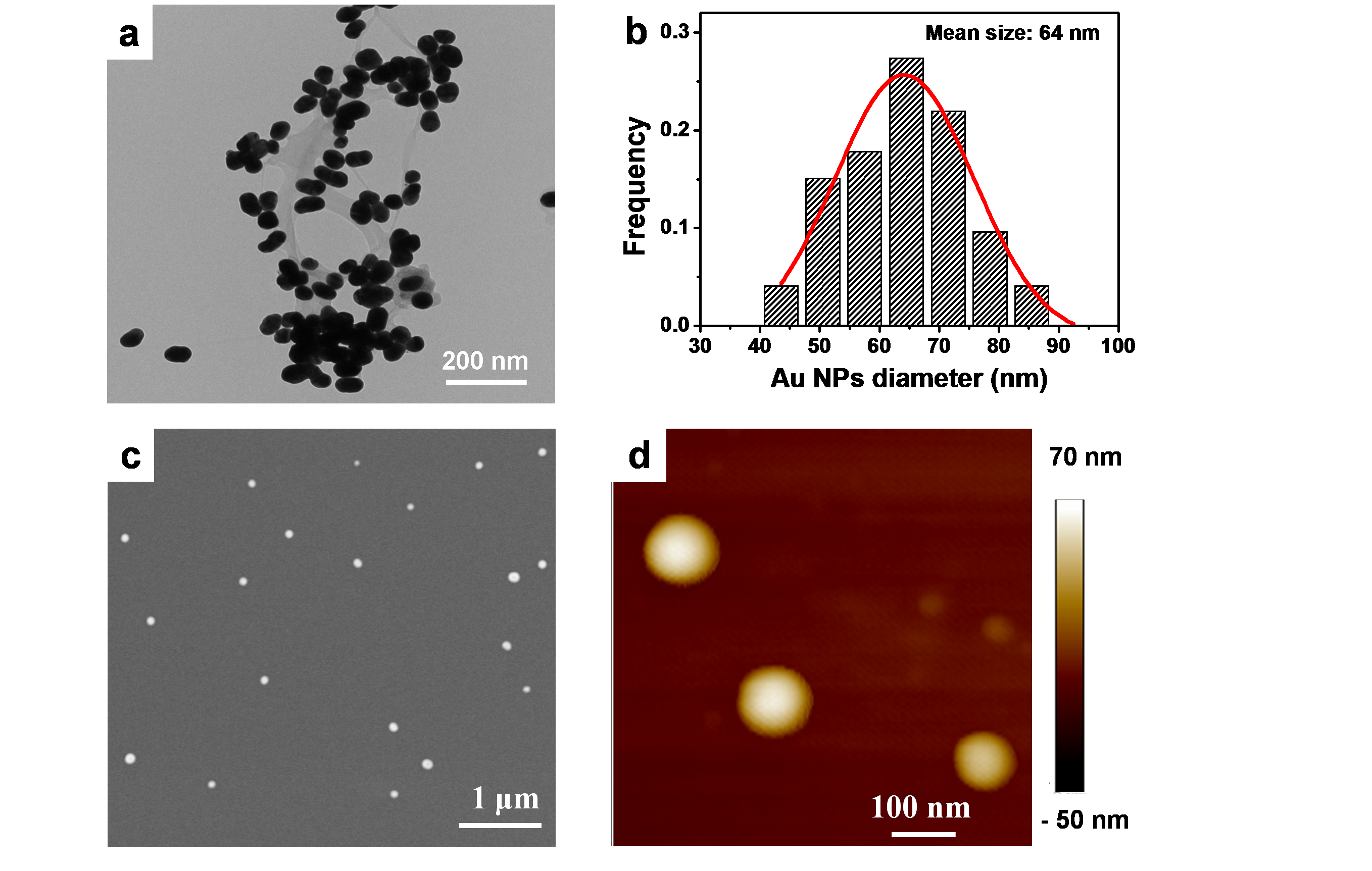
**

**Supplementary Figure 1.** Characterization of the prepared Au NPs and Au NPs/TiO_2_. (a) TEM image of the synthesized Au NPs. (b) Distribution of Au NP diameters, exhibiting an average size of 64 nm. The red line is a Gaussian fit. (c) SEM image of Au NPs on TiO_2_ substrate. (d) AFM image of Au NPs on TiO_2_ substrate. The Au NPs were dispersed on TiO_2_ crystal using a spin-coating method following by annealing. SEM and AFM images show that the monodisperse Au NPs are successfully deposited on the TiO_2_ substrate.


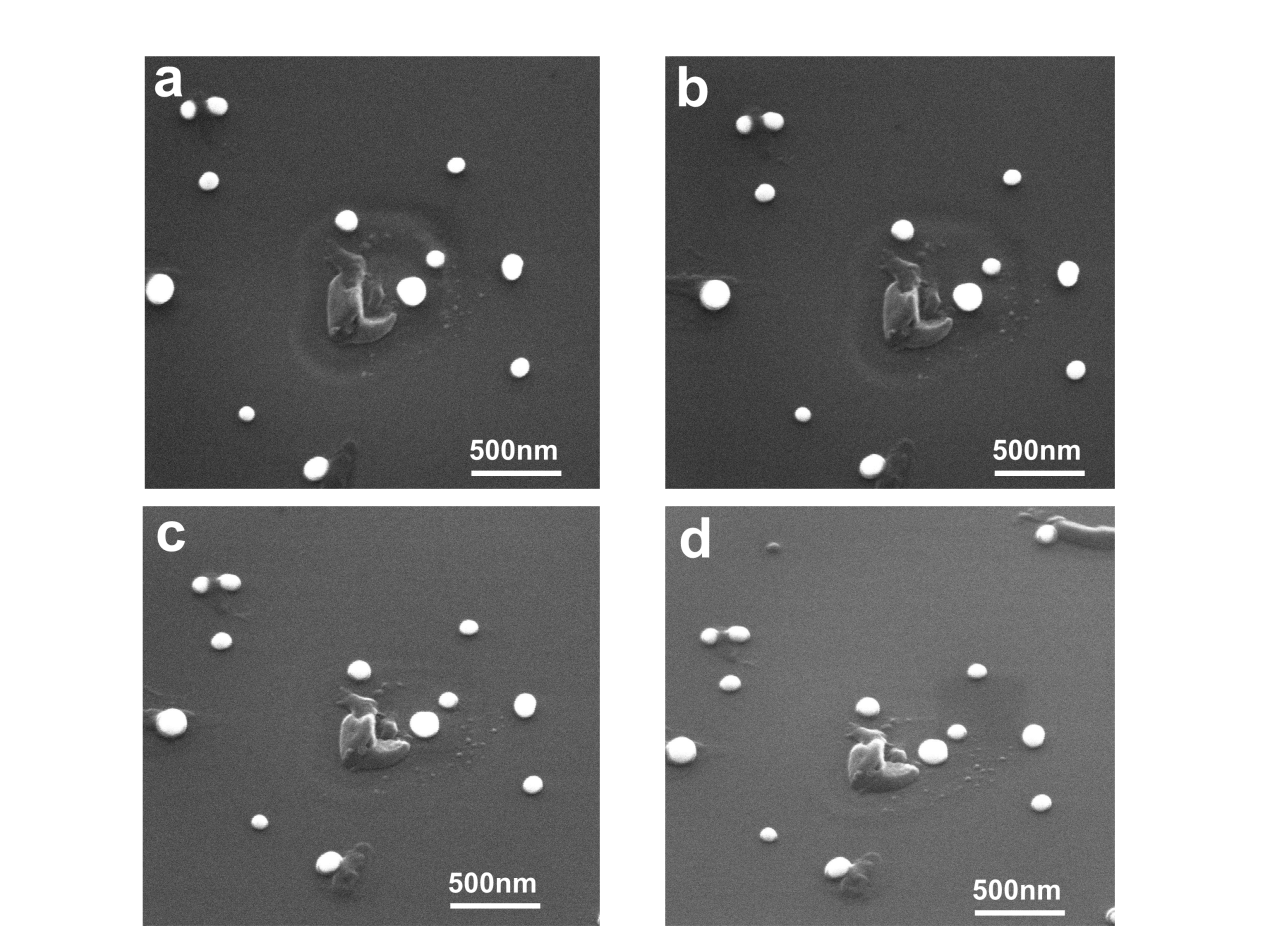


**Supplementary Figure 2.** Tilt-angle SEM of Au hemisphere/TiO_2_ nanostructure. SEM images observed at angles of 10 ° (a), 20 ° (b), 40 °, (c) and 50 ° (d). The results show that the Au NPs are in hemisphere shapes lying on the TiO_2_ substrate, yielding an intimate contact interface between Au and the TiO_2_ crystal. This exposed interface allows us to probe the surface potential at the Au/TiO_2_ interface.


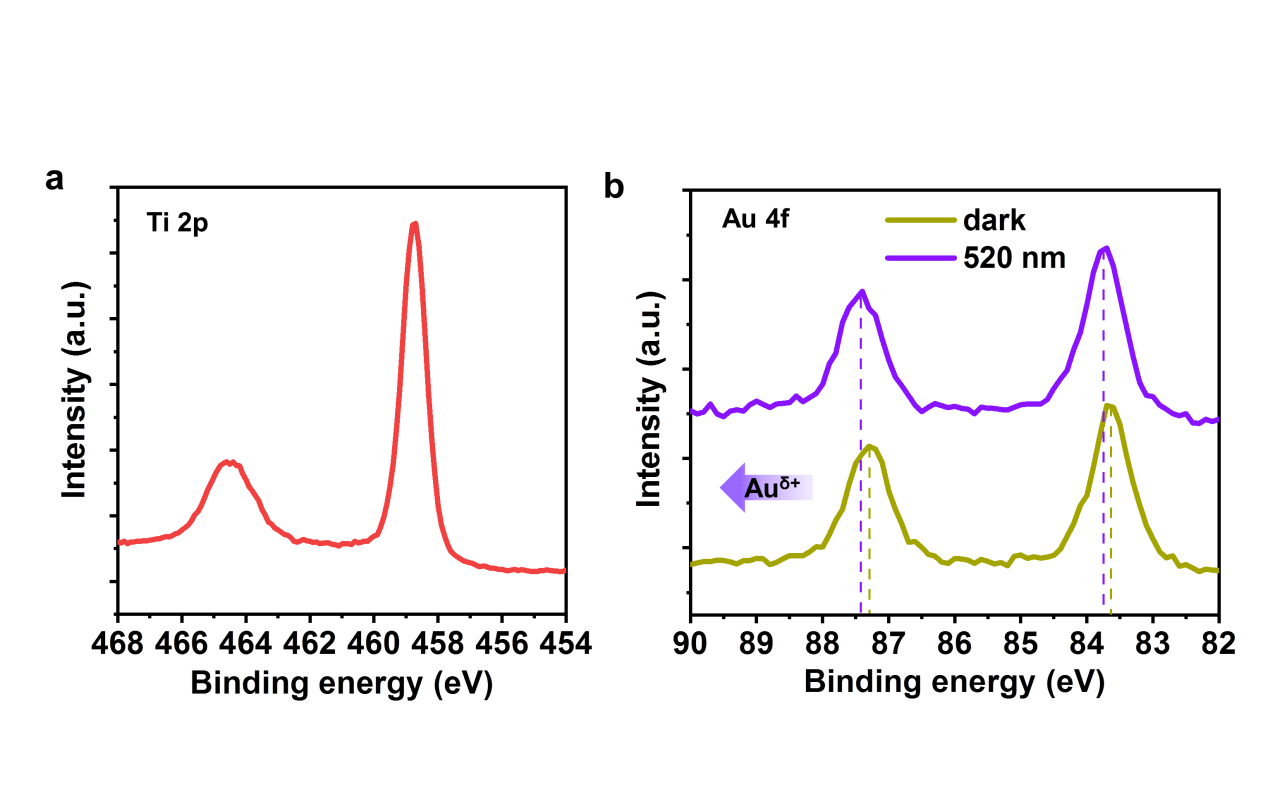


**Supplementary Figure 3.** XPS spectrum of Au/TiO_2_ sample. (a) Ti 2*p* XPS spectrum of Au/TiO_2_ sample. (b) Au 4*f* XPS spectrum of Au/TiO_2_ sample in the dark and under light illumination. The XPS of Au 4*f*_7/2_ in the annealed Au/TiO_2_ sample presents a peak at 83.7 eV, which has a small negative shift relative to 84.0 eV for bulk Au, indicating the intimate contact between Au and TiO_2_ [10].

**S3. Schottky barrier height measurement of Au/TiO_2_ using conductive atomic force microscope (C-AFM)**


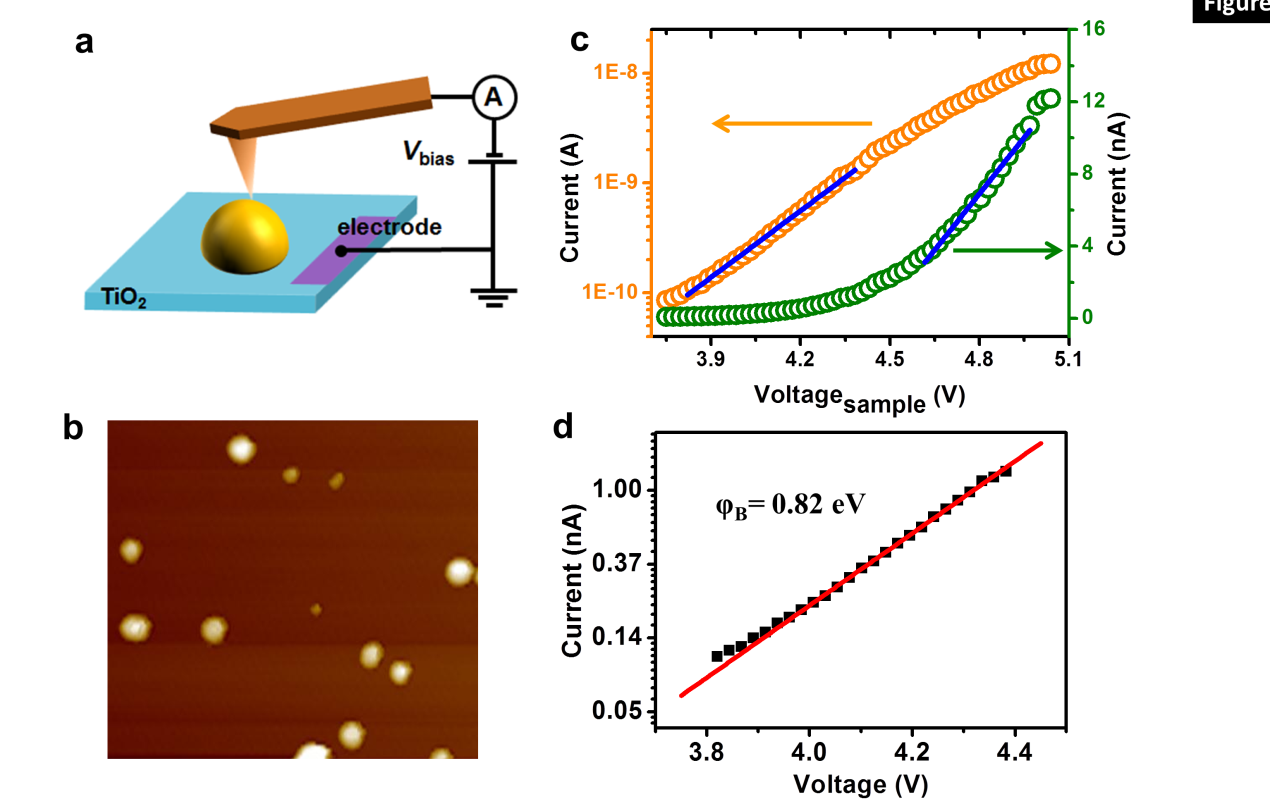


**Supplementary Figure 4.** C-AFM measurement of Au/TiO_2_ nanostructure. (a) Schematic of the experimental setup of C-AFM. (b) AFM image of Au/TiO_2_ sample using C-AFM mode. (c) Representative forward-bias *I–V* characteristic curve from C-AFM measurements, displayed both on a semi-logarithmic scale (left axis) and on a linear scale (right axis). (d) *I–V* curve of the Au/TiO_2_ nanostructure.

The C-AFM mode installed in the Bruker Dimension SPM system was used to study the sample *I–V* characteristics at the nanoscale. A topographic image was obtained and then the *I–V* curve was measured at the Au NP. Pt/Ir-coated probes (Bruker) were used for these measurements and the probe made contact with the sample surface in the *I–V* curve measurement.The *I–V* data were fitted to the thermionic emission equation described by [11,12]

$I={AA}^{*}T^{2}exp\left( -\frac{q\varphi_{B}}{kT} \right)exp\left[ \frac{q\left( V_{s}-IR \right)}{nkT} \right]$ (3)

where *A* = πr_tip_^2^ is the tip contact area, *A** =110 A cm^−2^ K^−2^ is the Richardson constant, *V_S_* is the applied bias voltage, *φ_B_* is the Schottky barrier height, *k* is the Boltzmann’s constant, *T* is absolute temperature (*T* = 297K), *q* is the electron charge, and *n* is the ideality factor. The resistance term *R* is the total resistance in this circuit, including the tip resistance, the charge diffusion resistance generated by the current to spread from a point contact to the Au/TiO_2_, the sample series resistance, and the resistance of the macroscopic contact.

At applied voltages exceeding the onset voltage in the sample, the *I–V* curve shows a linear relationship. The Schottky barrier *φ_B_* can be obtained from the semi-logarithmic plot in Supplementary Fig. 4c. The *φ_B_* is related to the intercept of the fitting linear relation. Finally, the Schottky barrier of Au/TiO_2_ interface is calculated as 0.82 eV. The barrier height obtained by our nanoscale analysis is in good agreement with reported values of *φ_B_* obtained by experimental measurement and theoretical simulation [13]. The photon energy of the Au NP SPR is larger than the Schottky barrier; thus, the plasmonic electrons with sufficient energy can be tranferred to TiO_2_. The strong electron scattering and electron-phonon scattering in Au NPs at the timescale of picoseconds results in the short lifetime of hot charges [14,15]. The interfacial charge transfer at Au/TiO_2_ can prolong plasmonic charge lifetime, which is important for charge-driven plasmonic photocatalysis.

**S4.** **KPFM measurements of Au/TiO_2_ nanostructure**


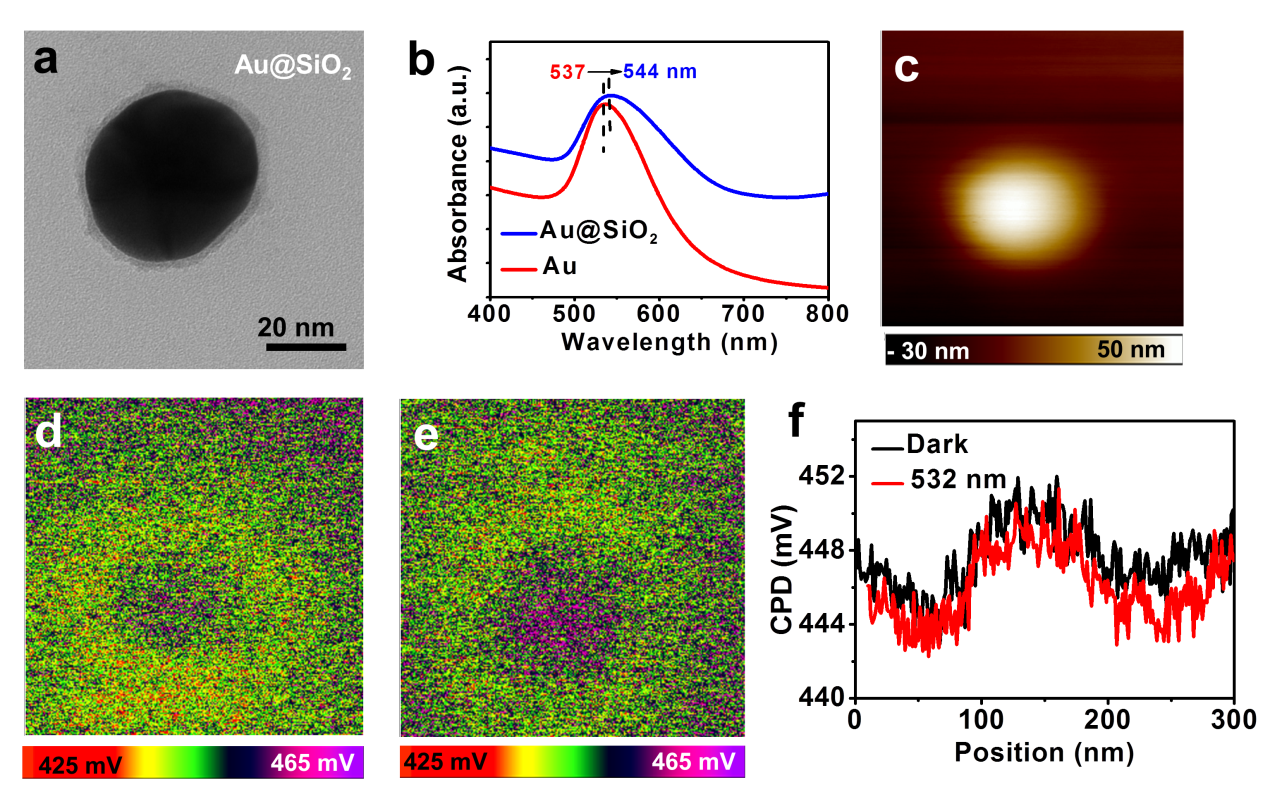


**Supplementary Figure 5.** KPFM images of Au@SiO_2_ NP/TiO_2_ nanostructure. (a) TEM image of Au@SiO_2_ NP. (b) Measured extinction spectra of Au NPs (red curve) and Au@SiO_2_ NPs (blue curve) in aqueous solution. (c) AFM image of Au@SiO_2_ NPs on TiO_2_ substrate. (d,e) Surface potential images of Au@SiO_2_ NP on TiO_2_ substrate in the dark (d) and under 532 nm light illumination (e). (f) Cross-section potential profile of Au@SiO_2_ NPs on TiO_2_ substrate extracted from surface potential image in the dark and upon visible light illumination.

The wavelength of the LSPR peak of the Au@SiO_2_ core-shell nanostructures shows a slight red-shift, consistent with previous reports [16]. The dark surface potential mapping of Au@SiO_2_/TiO_2_ shows the Au/TiO_2_ interface potential without any distinctive difference, because the interface is buried by the SiO_2_ layer. Moreover, it is interesting to find that the surface potential has no detectable change under SPR excitation. This result highlights the importance of direct interfacial contact for plasmon-induced charge spatial separation.


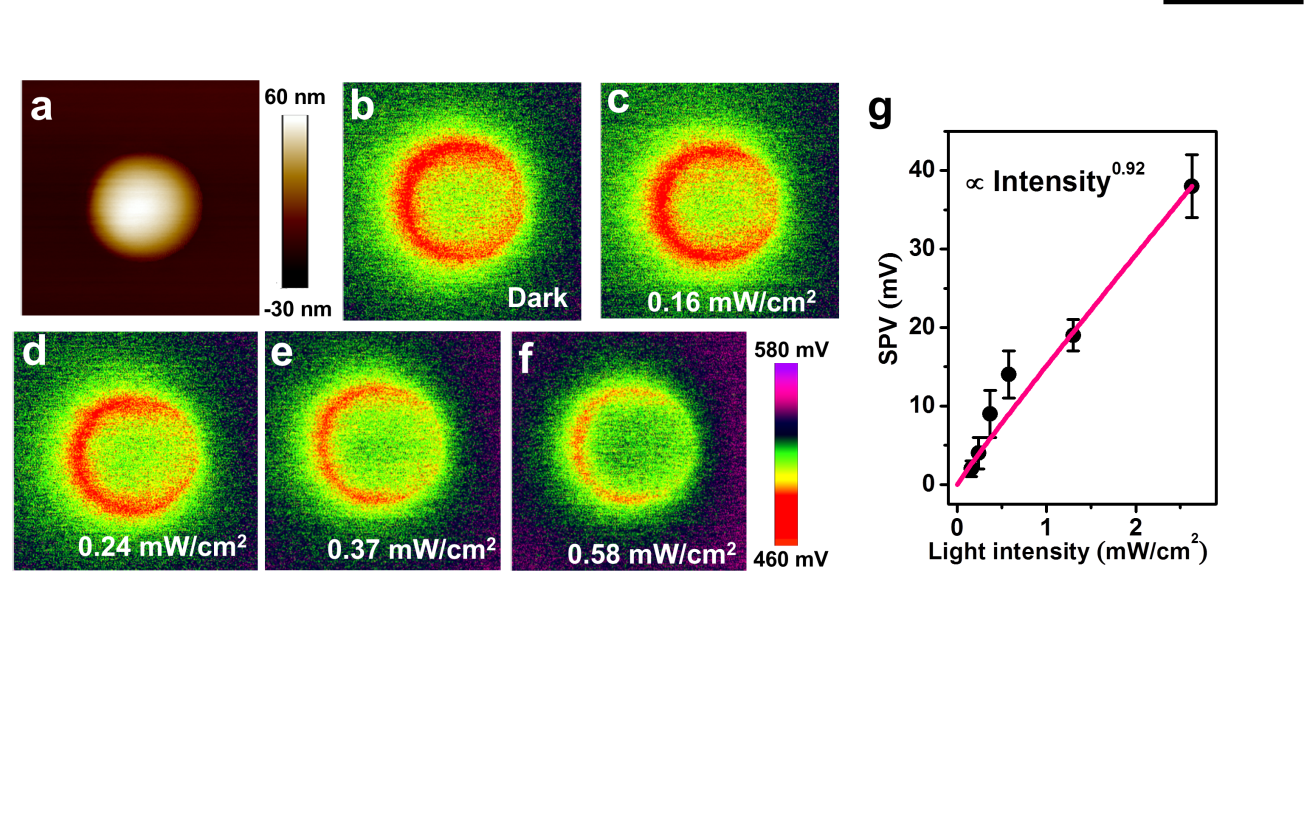


**Supplementary Figure 6.** SPV dependence of light intensity. (a) AFM image of Au/TiO_2_ sample. (b-f) Surface potential in the dark (b) and (c-f) upon 532 nm laser illumination with different light intensities. The asymmetric distribution of surface potential at Au/TiO_2_ interface can be ascribed to the different electrostatic force. (g) SPV plotted as a function of light intensity. Error bars are the relative deviations in the surface potential images.

It should be noted that the SPV can reflect the change in the charge density in the photoirradiation processes, and that only spatially separated photogenerated charges can produce local surface potential changes [17]. Thus, the magnitude of the positive SPV is directly linked to the number of residual holes. According to the measurement principle of surface potential, the SPV can be written as:

$SPV=Q_{s}\cdot\frac{d}{\varepsilon\varepsilon_{0}}$ (4)

where $Q_{s}$ is the hot holes density, d is a measurement parameter, $\varepsilon_{0}$ is the vacuum dielectric constant, and $\varepsilon$ is the dielectric constant of the sample. The number of SPR generated hot electrons/holes$Q_{g}$in relation to the illumination intensity can be expressed by the following formula:

$Q_{g}=A\cdot I$ (5)

where $Q_{g}$ is the number of Au NP SPR-induced hot holes, $A$ is a constant related to the absorption coefficient and quantum efficiency of Au NPs, and $I$ is the illumination intensity. Physically, an electron can be transferred to the contacting semiconductor only if it has sufficient kinetic energy to overcome the Schottky barrier while having a momentum within the injection cone [18], regardless of charge tunneling. The spatially separated hot hole/electron number $Q_{s}$ in Au/TiO_2_ with the Schottky barrier of $\Delta E_{B}$ can be well approximated by the results from the Fowler theory [13,18]:

$Q_{s}=S_{\mathrm{probability}}\cdot Q_{g}=c\cdot Q_{g}\cdot\left( hv-\Delta E_{B} \right)^{2}$ (6)

where $S_{\mathrm{probability}}$ is the probability of hot electron injection to the TiO_2_ semiconductor and $c$ is a constant. Thus, the measured SPV is proportional to the incident light intensity. As expected, the experimental result indeed shows that the SPV at the Au/TiO_2_ interface exhibits approximately linear (first-order) dependence on the light intensity (Supplementary Fig. 6). This confirms that the surface potential change occurs via a linear process. Thus, the observed enhanced SPV at nanogap of Au dimer/TiO_2_ can be ascribed to the strong optical field due to plasmon coupling.


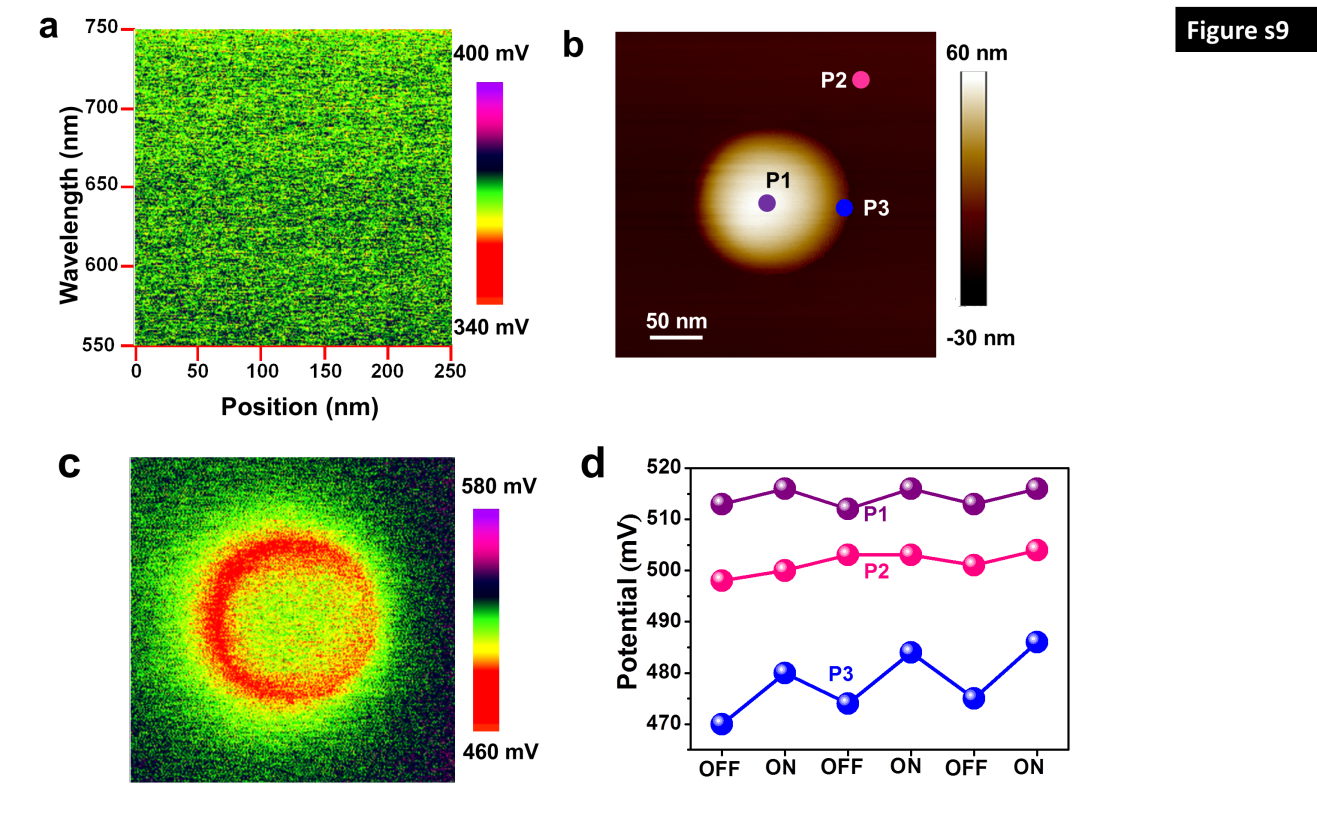


**Supplementary Figure 7.** Surface potential measurements on rutile (100) crystal surface and Au/TiO_2_ nanostructure. (a) Surface potential image of rutile (100) crystal under visible light irradiation. (b) AFM image of Au NPs/TiO_2_. (c) Surface potential image of Au NPs/TiO_2_. (d) Surface potential measured at Au surface (P1), TiO_2_ surface (P2) and Au/TiO_2_ interface (P3), as indicated in b, when the light of 550 nm is turned on and off.

Since the surface potential of the TiO_2_ crystal has no perceptible change under visible-light illumination (Supplementary Fig. 7a), the possibility of intraband states or surface states of TiO_2_ contribution to photo-induce surface potential change can be excluded. In addition, clear reversible changes of surface potential recorded at the Au/TiO_2_ interface are observed when switching the light on and off, signifying that the surface potential change caused by the plasmonic charge interfacial transfer.

The most striking feature of the surface potential change is mainly generated at the Au/TiO_2_ interface. The increased potential indicates holes localized at the interface, while electrons are injected into TiO_2_. Due to the upward band bending of TiO_2_, the injected electron migrated into the bulk of TiO_2_, leading to a small surface potential change. Nevertheless, the SPV at Au/TiO_2_ interface induced by plasmonic charge interfacial transfer is small compared to the SPV signal of general semiconductors. We consider at least three reasons contributed to this phenomena. First, in physics, an electron can be transferred to a contacting semiconductor only if its momentum is within the injection cone [13,18], despite the energetic plasmonic charge generated in Au NPs [19]. Second, after generating plasmonic electrons inside the metal, they travel and scatter with other electrons and phonons, and only a fraction of the electrons can reach the metal–semiconductor interface. The plasmonic charge interfacial injection will compete with the thermalization of the charge population [20]. Third, plasmonic electron generation is not the only decay channel of surface plasmon energy [15]. All these intrinsic factors create restrictions for the number of spatially separated plasmonic charge.


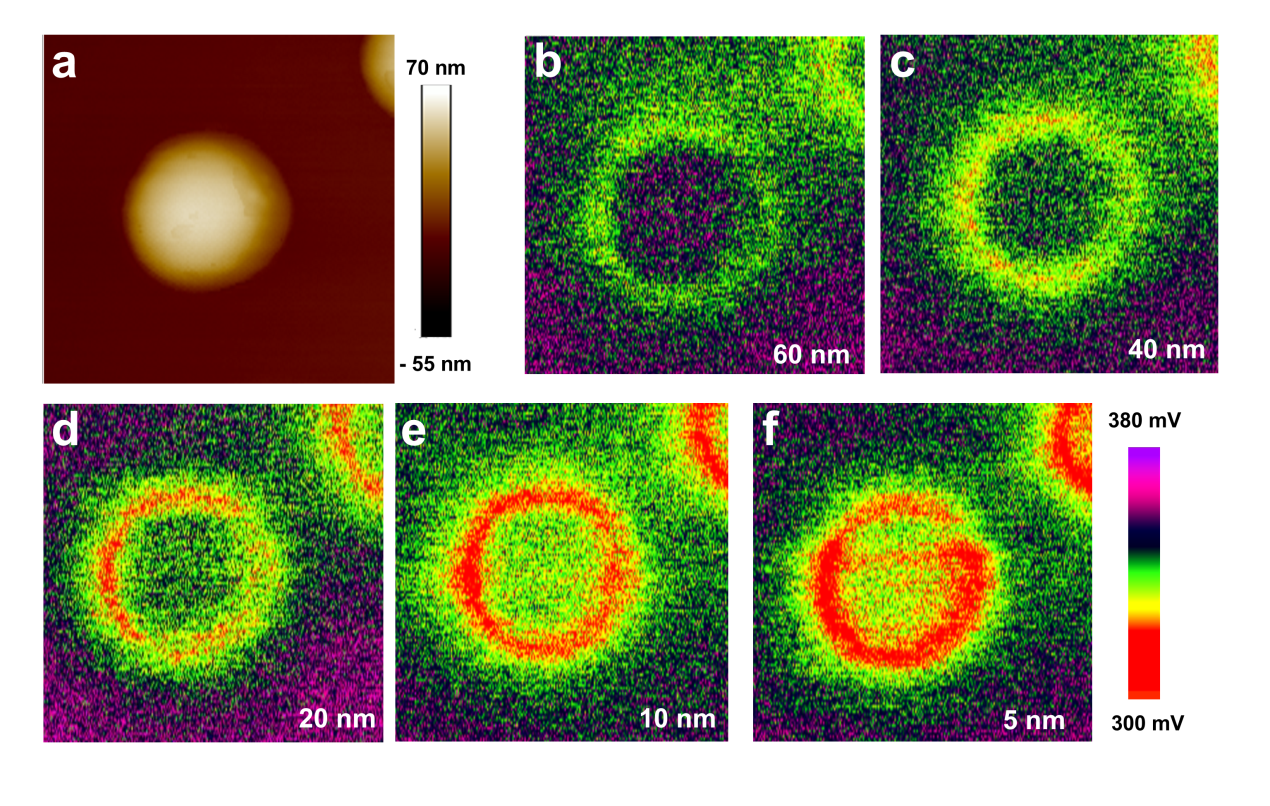


**Supplementary Figure 8.** KPFM images of Au/TiO_2_ nanostructure recorded at different lift heights. (a) AFM image of Au/TiO_2_ nanostructure. (b-f) Surface potential image of Au/TiO_2_ nanostructure measured at different lift heights: (b) 60 nm, (c) 40 nm, (d) 20 nm, (e) 10 nm, and (f) 5 nm.

With decreasing tip–sample distance, the Au/TiO_2_ interface potential becomes more pronounced. This is because the KPFM measured potential is a weighted result from a much larger effective area than the tip apex [21]. Since the height of the truncated cone of the tip (10–15 μm) is larger than the tip–sample distance (10 nm), the interaction between the tip apex and the sample is more sensitive to tip–sample distance compared to the interaction between the cantilever and sample. Reducing the tip–sample distance can improve the spatial resolution and accuracy of the surface potential [21-23]. However, the small tip–sample distance also results in unstable potential signals and poor repeatability, especially at the Au/TiO_2_ interface because of the rapid probe lifting process. To minimize the surface potential signal noise and obtain high-quality images, the probe lift height of 10 nm is used in the experiments.


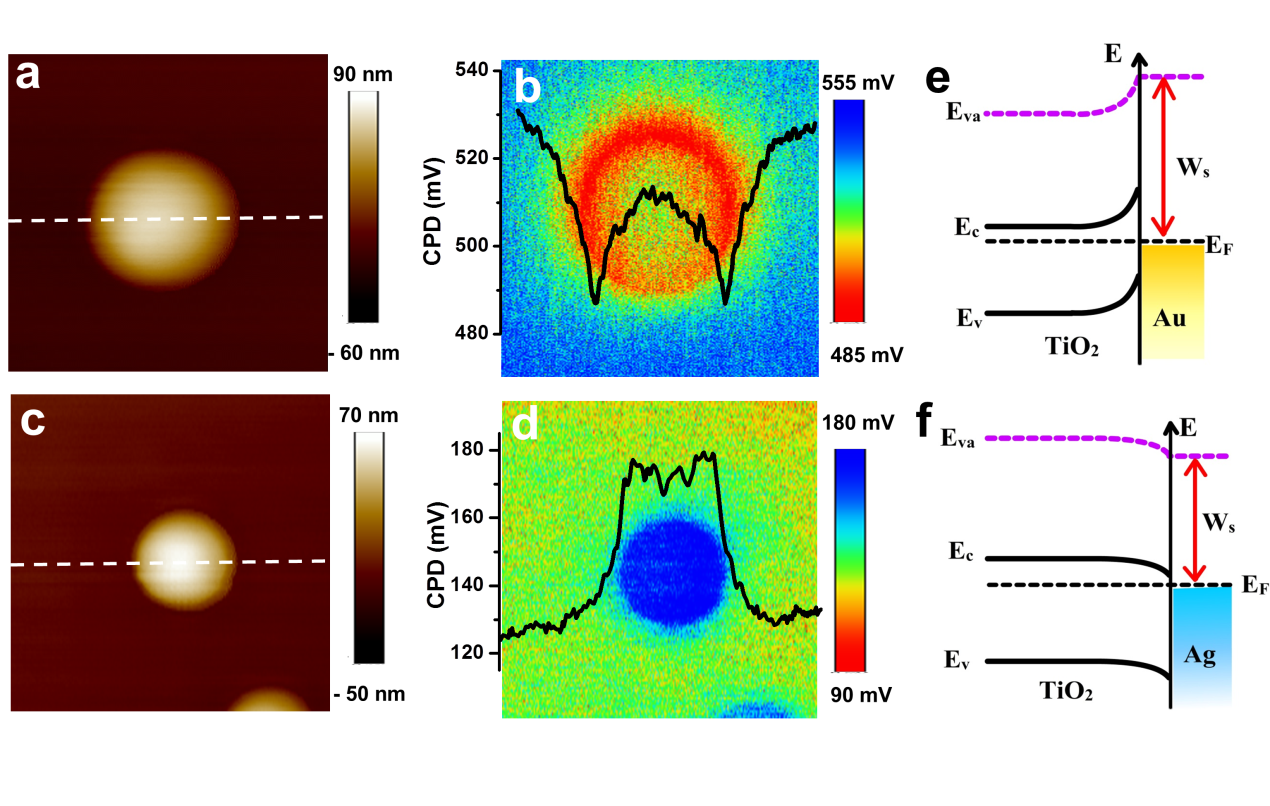


**Supplementary Figure 9.** KPFM measurement of Au/TiO_2_ and Ag/TiO_2_. (a,b) AFM image (a) and surface potential image (b) of Au/TiO_2_. (c,d) AFM image (c) and surface potential image (d) of Ag/TiO_2_. The surface potential line profiles in b, d are extracted across the center of particle, as indicated with white line in a, c, respectively. (e,f) The band diagram for the Au/TiO_2_ (e) and Ag/TiO_2_ (f).

Because the different SCM-PIT tips have been used to measure surface potential of Au/TiO_2_ and Ag/TiO_2_ sample, the work function of different tips were calibrated with highly oriented pyrolytic graphite (HOPG). The work functions of the Au and Ag NPs were measured to be 4.83 and 4.56 eV, respectively, which are in the range of the literature values [24]. The surface potential of Au/TiO_2_ displays a considerable difference at the interface, which may originate from either geometrical effects, cross-talk effects between the topographic and potential channels, or work function differences from the interfacial Schottky junction. The tip geometrical effect is not likely to be the case because the lower surface potential at the interface is not affected by the tip shape (Supplementary Fig. 10). To clearly determine the origin of surface potential difference, we further performed KPFM measurements for Ag NP/TiO_2_ crystal (the size of the Ag NP is comparable with that of the Au NPs). Au NP and Ag NP have the same morphologies, but they have different work functions. Interestingly, the surface potential image of Ag/TiO_2_ is significantly different from that of Au/TiO_2_. The surface potential at the Ag/TiO_2_ interface is pronouncedly higher than that of the TiO_2_ surface, but is slightly higher than that of the Ag surface (Supplementary Fig. 9d). This behavior can be attributed to the downward energy-band bending because of the lower work function of Ag compared to that of the used TiO_2_ crystal (work function: 4.65 eV, HOPG). These results suggest the negligible cross-talk effects between the topographical image and the surface potential channels in the dual-pass scan process. This verifies that the local contrast of surface potential originates from the work function differences at the interface.


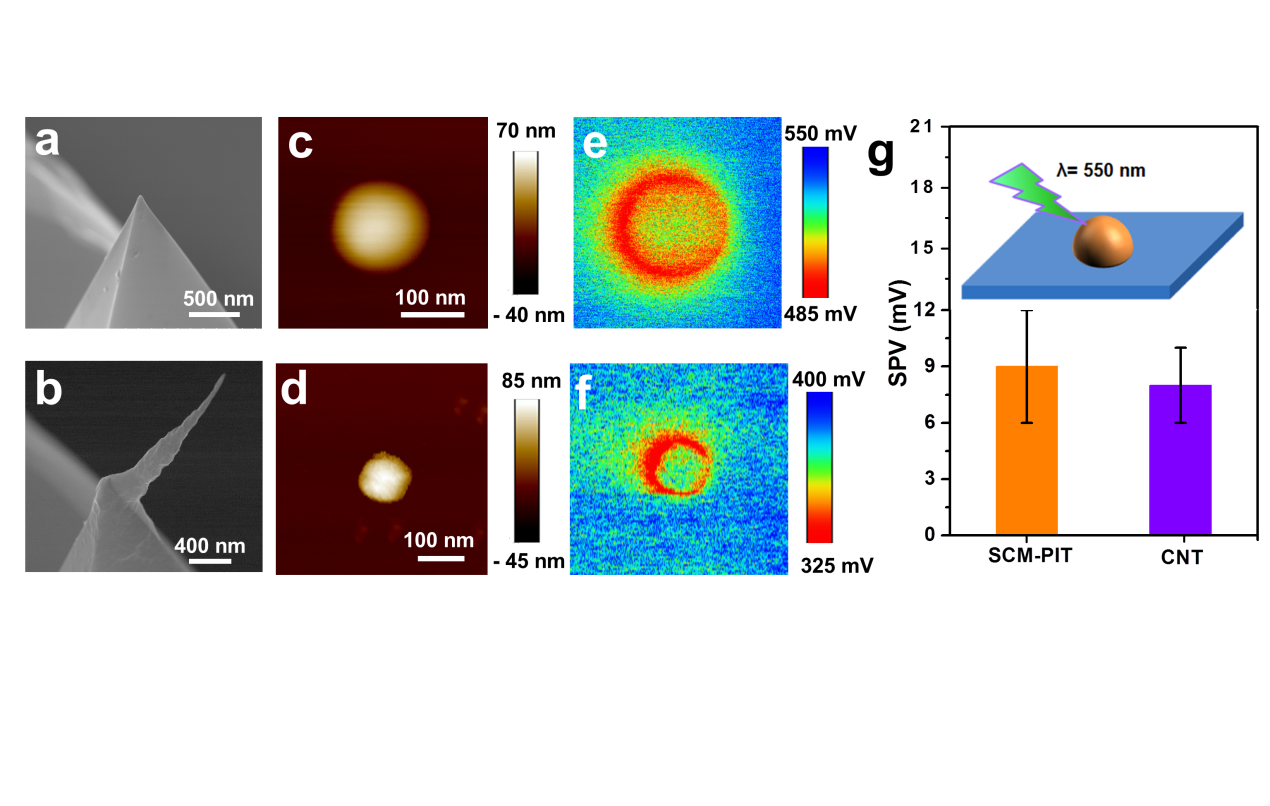


**Supplementary Figure 10.** KPFM measurements of Au NP/TiO_2_ nanostructure with different probes. (a,b) SEM images of SCM-PIT probe (a) and anchored carbon nanotube (CNT) conductive probe (b). (c-f) Topographic images (c,d) and surface potential images (e,f) of Au/TiO_2_ in the dark using SCM-PIT and CNT probe, respectively. (g) SPV comparison of Au/TiO_2_ when illuminated at 550 nm (2 mW/cm^2^). The inset is the schematic of the illuminated Au/TiO_2_.

Compared to the SCM-PIT tip, the CNT tip (resonant frequency: 58 kHz) has nearly the same cantilever dimensions but a smaller radius (5 nm) for the tip apex and a long CNT (~ 1.5 μm) at the apex of silicon probe. This typical morphology weakens the weighted effect of the surface potential and leads to a higher spatial resolution for the CNT probe. However, a lower potential is also observed at the Au/TiO_2_ interface using the CNT probe, which also illustrates that the potential distribution originates from the work function difference rather than from geometrical effects. In addition, for the nearly identical Au NP, the SPV variation caused by tip morphology is within experimental error, demonstrating the negligible effect of probe shape on the SPV measurement.


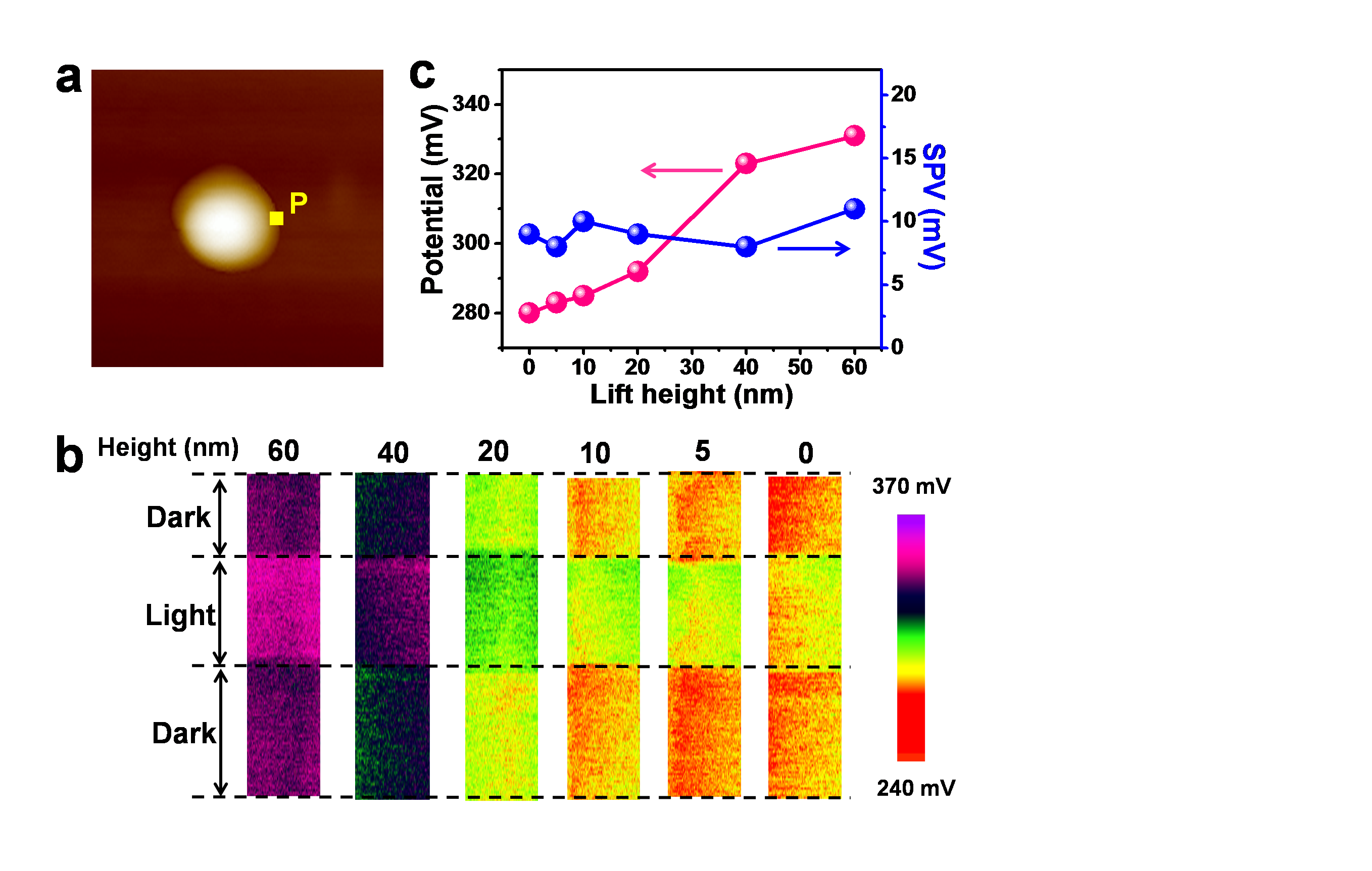


**Supplementary Figure 11.** Lift height effect on surface potential and SPV at Au/TiO_2_ interface. (a) AFM image of Au/TiO_2_ nanostructure. (b) Surface potential image at Au/TiO_2_ interface in the dark and under 560 nm illumination recorded for different lift heights at the same location. The measured position is marked by a yellow bulk in a. (c) Potential and SPV as a function of lift height.

According to our foregoing discussion, decreasing the tip–sample distance can increase the spatial resolution of the surface potential because it decreases averaging effects. This behavior is corroborated for surface potential measurements at different tip–sample distances, as the interfacial surface potential is dependent on the lift height in lift mode. However, the fluctuations of the SPV at the same location for different lift heights are less than 4 mV, suggesting that the lift height has negligible effect on the SPV measurement in our case. We thus focus on analyzing the SPV to determine plasmonic charge spatial separation.

**S5. Geometry deconvolution procedures**

In order to retrieve the actual interparticle separation distance in the Au dimers from the measured AFM topography, the profile of Au dimers required deconvolution. According to the AFM profiles of Au NPs dimers of different configurations, it was possible to retrieve the real geometrical parameters of an Au NP dimer through a simple calculation [25].


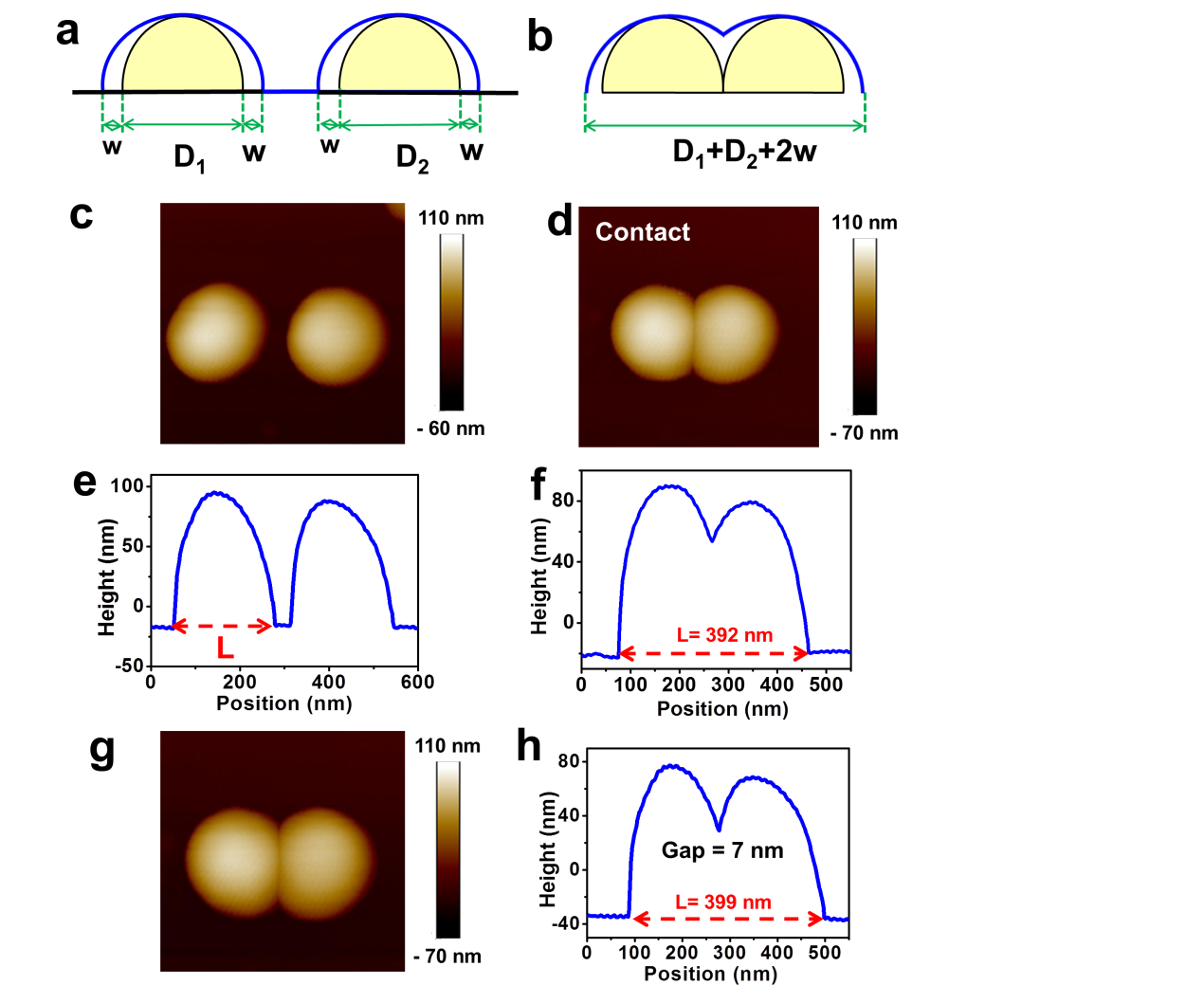


**Supplementary Figure 12.** Procedure for obtaining accurate separation distance of Au dimer. (a,b) Structural model of separated Au NPs on TiO_2_ (a) and Au NPs placed close together on TiO_2_ substrate (b). w is the broadening caused by the tip-sample convolution effect. (c,d) AFM images of separated (c) and (d) contact two Au NPs. (e,f) Height profiles (e) and (f) derived from c and d, respectively. (g) Topography of Au dimer with small gap on TiO_2_ substrate. (h) Height profile of Au dimer derived from g.

The actual Au dimer separation distance is larger than the measured dimension extracted directly from the AFM morphology. As shown in Supplementary Fig. 12a,b, the solid blue lines are the measured AFM height profiles, and the yellow zone is the hemispheric Au NP. The widening of an individual particle from the contribution of tip-sample convolution is 2 × w. It is clearly seen that the AFM image profiles are convolutions of the sample and tip geometries, which yields correct heights but overestimated widths for the Au particles. To retrieve the real distances of the Au dimers, we manipulate the Au dimer to obtain larger distances (c) and smaller distances (d) for the same two particles. We assume that the particles do not deform when they are removed and brought together. Thus, the measured lateral size L in Supplementary Fig. 12e is given by D_1_+2w and *L* in Supplementary Fig. 12f is given by D_1_+D_2_ +2w. This procedure allows us to obtain the real distances of the Au dimers described in main text. AFM images show that the D_1_ + D_2_ + 2w = 392 nm for contact two NPs in Supplementary Fig. 12f, while the D_1_ + D_2_ + gap + 2w = 399 nm for Au dimers in Supplementary Fig. 12h. Thus, the nanogap size of Au dimer is 7 nm.

**S6. Evaluation the photothermal effect of Au NDs/TiO_2_**

The surface potential of the sample depends appreciably on the testing temperaturein semiconductors [26]. The photothermal heating of Au NPs producesa hot surface through electron–phonon thermal equilibrium under illumination at the resonance wavelength, which leads to a static electrochemical potential [27]. Upon SPR excitation, the thermal response depends on the light power and nanoparticle size. The change of surface temperature on sample can be calculated based on a steady-state condition [28]. The temperature change is given by

$\Delta T=\frac{Q}{4\pi kr_{NP}}$ (7)

where $Q$ is the heat generated by the Au NP, *k* is the thermal conductivity of the Au, and $r_{NP}$ is the radius of the Au NP. The amount of heat relates to the absorption cross-section area of Au and the light power, and can be expressed as:

$Q= C_{abs}\times I$ (8)

where $C_{abs}$= 3.8 $\times{10}^{-14}m^{2}$ is the absorption cross-section of Au and the light power (*I*) used is 2 mW/cm^2^. When$r_{NP}$is 70 nm, the photothermal effect yields the temperature change $\Delta T$ = 1.4 μK on the Au particle surface after Au SPR excitation. For Au ND/TiO_2_ sample, we have evaluated the photothermal effect at hot spot. According to the results of calculated near-field image, the photothermal effect induced the temperature change at hot spot is 256 μK after SPR excitation. Although the value of SPV is sensitively dependent on the sample temperature [29], this minor temperature variation effect on SPV is negligible in this case.

**S7. KPFM measurements and electromagnetic field simulation of Au dimer/TiO_2_**


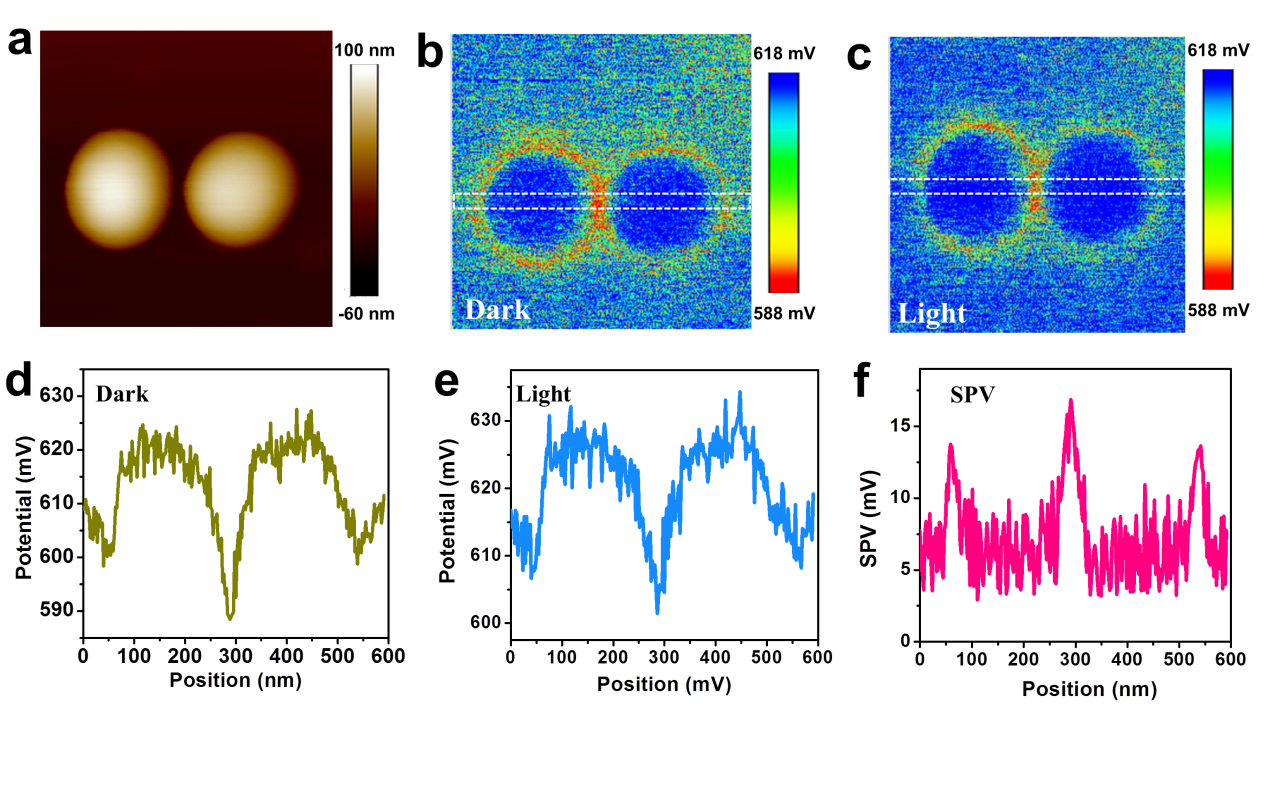


**Supplementary Figure 13.** KPFM images of Au ND/TiO_2_ nanostructure. (a) Topography of Au ND/TiO_2_ nanostructure. (b,c) Surface potential images of Au ND/TiO_2_ in the dark and under light illumination. (d-f) Potential distribution line in the dark (d), Potential distribution line under light illumination (e) and SPV distribution line (f) of Au ND/TiO_2_ nanostructure. Potential distribution line is the average results as indicated by dotted line in b,c.


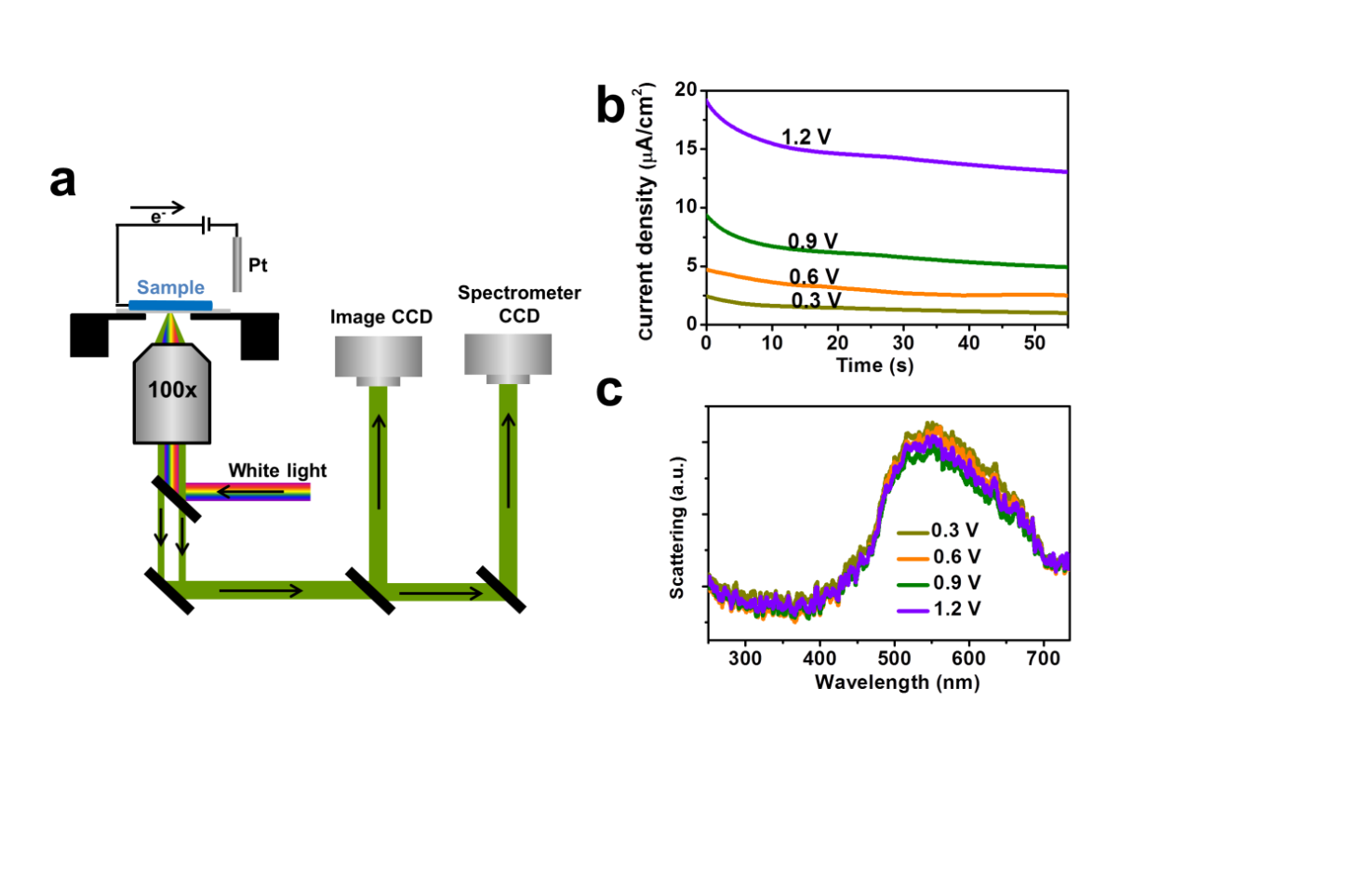


**Supplementary Figure 14.** (a) Schematic of the dark field microscopy and two electrodes measurement setup. (b) current of Au dimers/TiO_2_ sample at different applied voltage. (c) Scattering spectroscopy of Au dimers/TiO_2_ sample at different applied voltage.


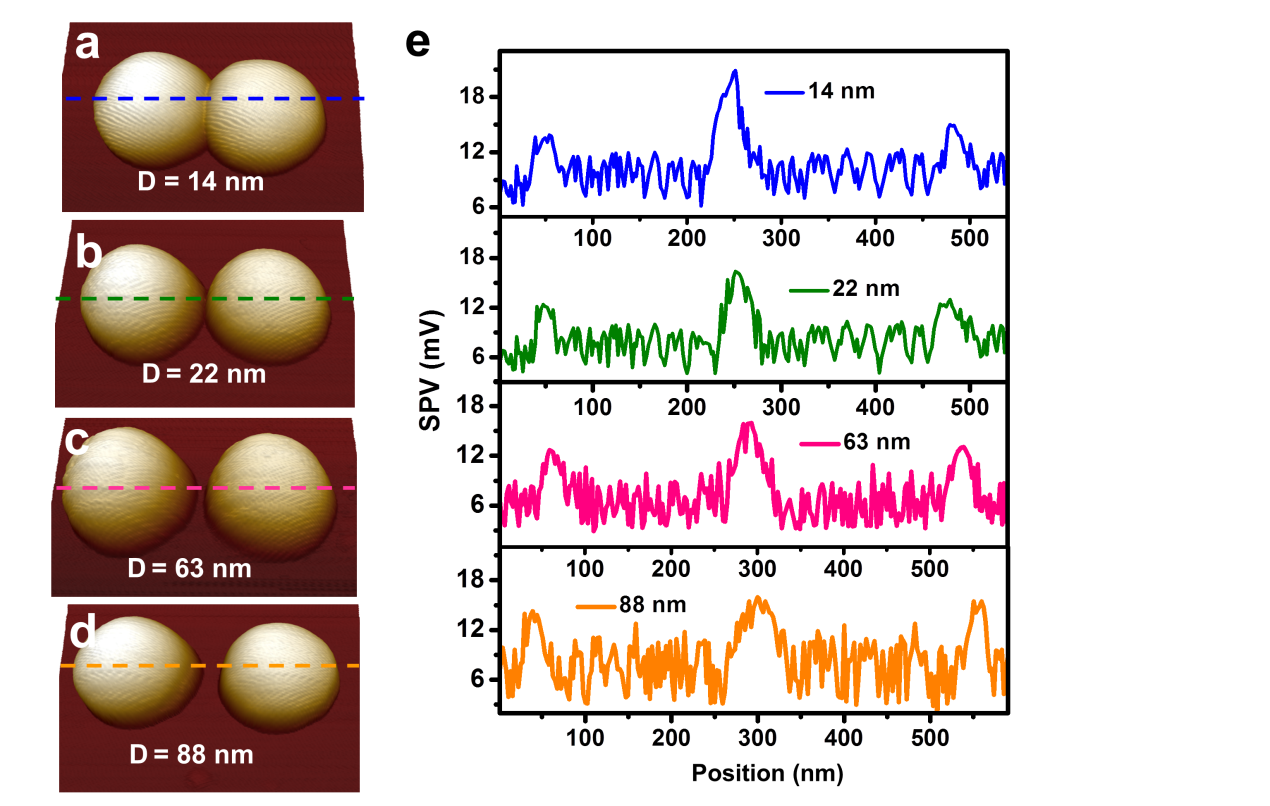


**Supplementary Figure 15.** SPV of Au NPs dimer/TiO_2_ under light polarization of 90°. (a-d) 3D AFM images of different Au dimers on TiO_2_ substrate. (e) SPV profiles of Au dimer/TiO_2_ along dimer axis, as marked in AFM images. The SPV of Au dimer/TiO_2_ was recorded at the peak of the surface potential spectrum (Figure 2c in the main text). The light polarization is parallel to the dimer axis.

The interparticle distance (D) of the Au dimer can be controlled with a range from several nanometers to dozens of nanometers. We observe almost the same SPV values of the side spots at the outer ends of the Au dimers after coupled SPR excitation. However, the SPV at the nanogap is significantly increased with decreasing interparticle separation distance. This result indicates that strong plasmon coupling leads to the high density of spatially separated charges at interface within nanogap of Au dimers/TiO_2_ nanostructures.


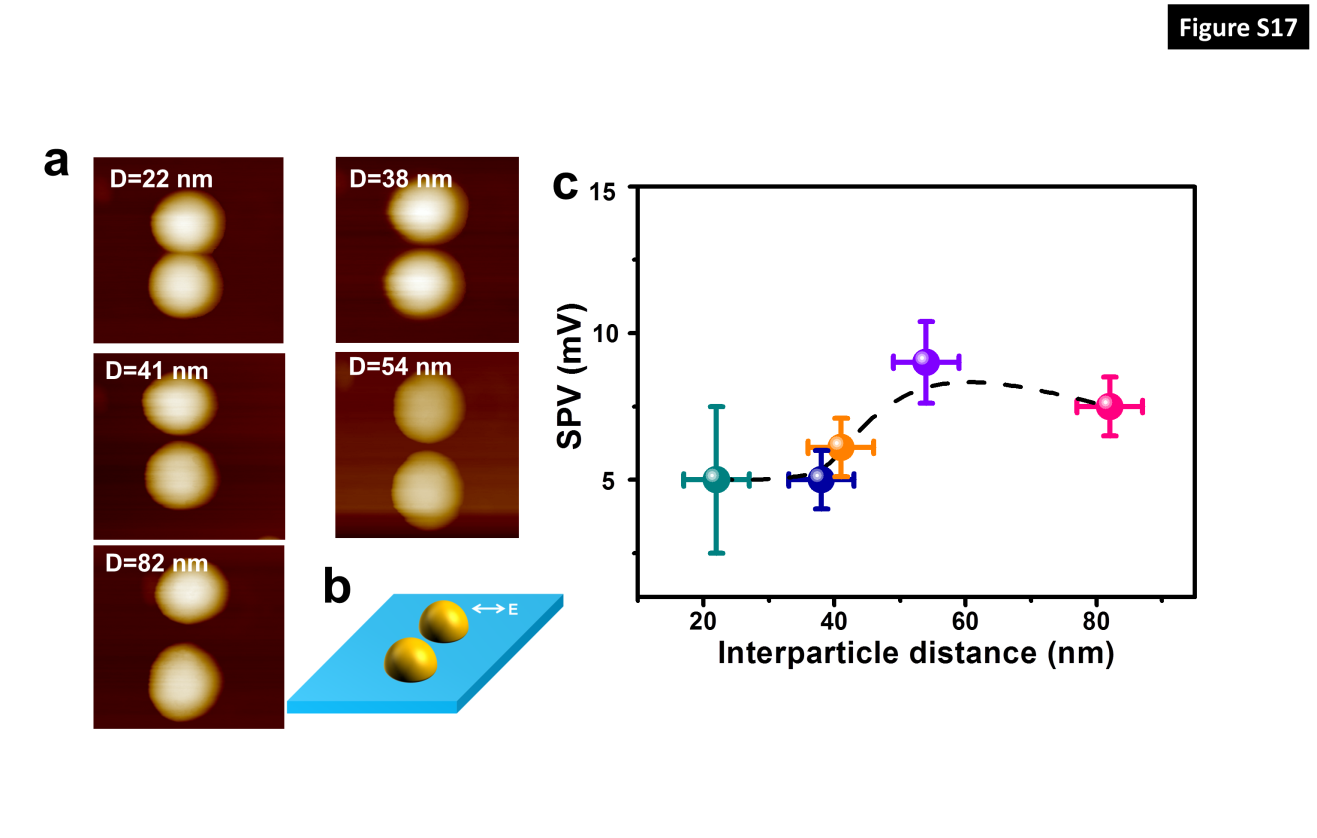


**Supplementary Figure 16.** SPVmeasurement of Au dimer on TiO_2_ substrate under light polarization of 0 °. (a) AFM images of Au dimers/TiO_2_ varying separation distances. (b) Schematic of the light polarization for SPV measurements. (c) SPV at nanogaps of Au dimers with different separation distances. Error bars of the SPV and interparticle distance represent relative deviations in KPFM images.


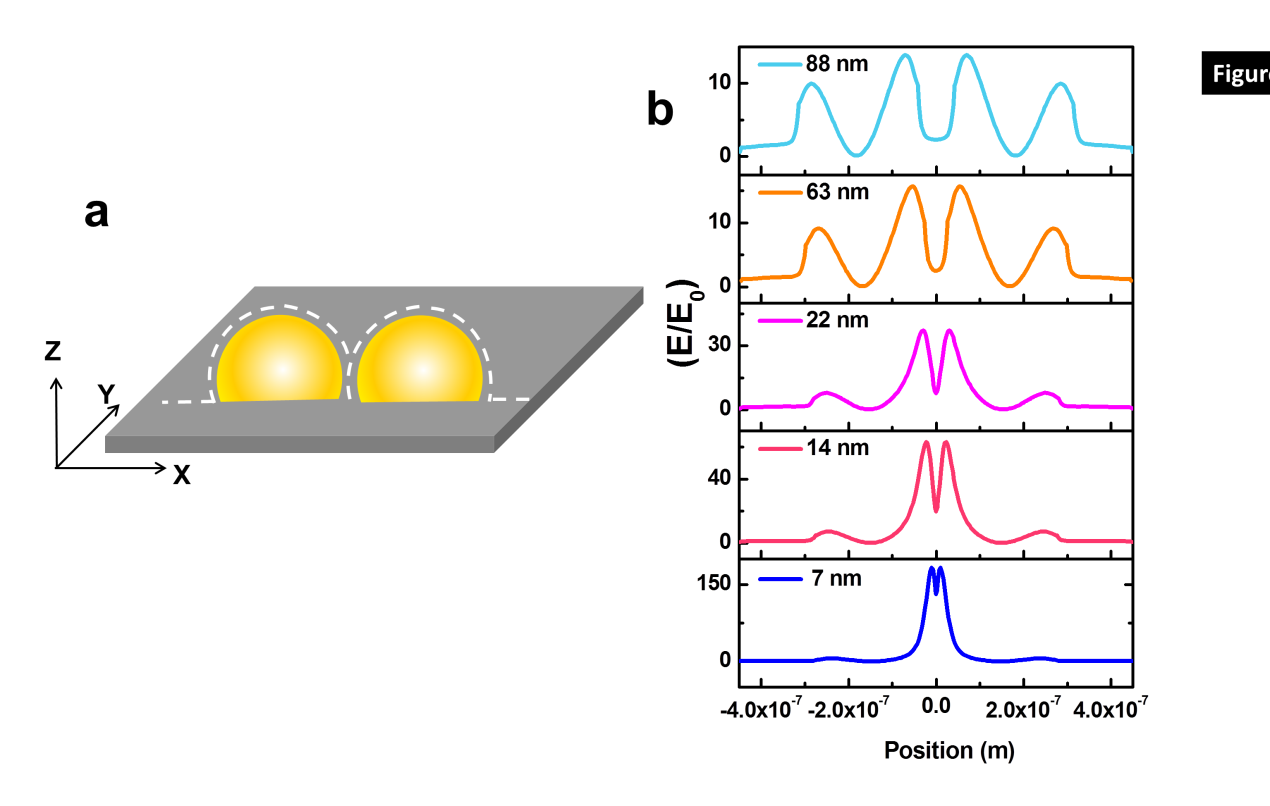


**Supplementary Figure 17.** Simulated line profiles of near-field enhancement for Au dimer/TiO_2_ nanostructure. (a) A schematic depicting the location of line profile data in Au dimer/TiO_2_ nanostructure. (b) Near-field intensity enhancement of Au dimer/TiO_2_ nanostructures with different interparticle distances as a function of distance along the dashed line indicated in a. Note that the incident light is polarized along the axis of the dimer (X-axis).

The magnitude of near-field enhancement is governed by the distance between the two Au NPs. With the decrease of interparticle distance, the near-field intensity enhancement at the nanogap is greatly enhanced, but that at the side spots at the outer ends of the Au dimer is increased only slightly. This illustrates that the near-field intensity between neighboring Au NPs can be significantly enhanced by concentrating incident beams into a narrow nanogap [30].

**S8. Calculating plasmonic holes** **density of Au dimer/TiO_2_ nanostructure**

After Au SPR excitation, hot electrons can be injected into the TiO_2_ semiconductor. The number of hot holes left at the Au dimer/TiO_2_ interface is quantitatively estimated using the general equation for a spherical capacitor [24,31]:

$N_{separated holes}=\frac{C}{e}\mathrm{SPV}$ (9)

where $C$ is the tip–sample capacitance on the Au NP and *e* is the absolute value of the elementary charge. The capacitance probed by the nanometric tip apex on the Au/TiO_2_ is given by [24]

$C=4\pi\varepsilon_{R}\varepsilon_{0}R$ (10)

where $\varepsilon_{0}$is the vacuum permittivity, $\varepsilon_{R}$= 70 is the relative permittivity of rutile (100) crystal [32], and $R$ = 25 nm is the sphere radius of the tip. For the Au dimers/TiO_2_, the restored SPV is shown in the main text (Figure 1c). We can extract the number of spatially separated hot holes as 25.5 at Au dimers/TiO_2_ interface in the region of nanogap and 3.8 at the outer ends of the Au dimers/TiO_2_ interface. In our current SPV measurement, when the tip is 10 nm above the surface of sample, the SPV resolution (*r*) in the restored SPV profile is ~45 nm for the Au dimer/TiO_2_. We can estimate the local density of separated holes at Au dimers/TiO_2_ interface in the region of nanogap by assuming that the tip can sense a circular sample region of 45 nm in diameter. The density of separated holes is:

$n=N_{separated holes}/\pi r^{2}$ (11)

The density of separated hot holes at Au dimers/TiO_2_ interface in the region of nanogap is calculated as 4 × 10^−3^ nm^−2^, which is a high density for photogenerated hot holes under low light density (0.5 mW/cm^2^). Similarly, the density of separated hot holes at Au NPs/TiO_2_ interface is calculated as 6 × 10^−4^ nm^−2^.

**S9. Plasmonic Au NP/TiO_2_ and Au ND/TiO_2_ photoanode**

**
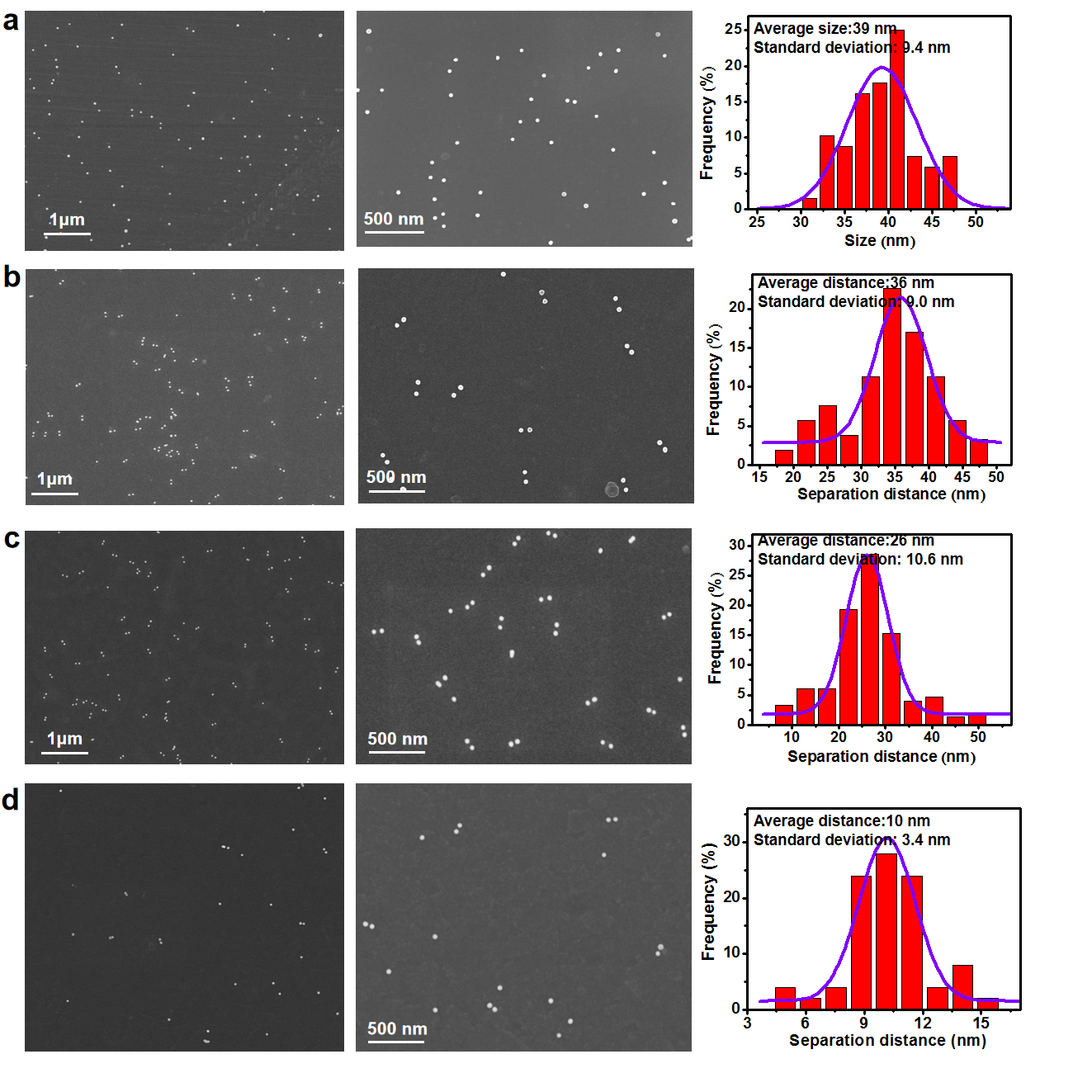
**

**Supplementary Figure 18.** Morphology and size distribution of Au NP/TiO_2_ and Au ND/TiO_2_ sample. (a) SEM images of Au NP/TiO_2_ and size distribution of the Au NPs by analysing the SEM images. The mean size of Au NPs was 39 nm. (b) SEM images of Au ND/TiO_2_ and separation distance distribution of the Au NDs by analysing the SEM images. The average interparticle distance was 36 nm. (c) SEM images of Au ND/TiO_2_ and separation distance distribution of the Au NDs by analysing the SEM images. The average interparticle distance was 26 nm. (d) SEM images of Au ND/TiO_2_ and separation distance distribution of the Au NDs. The average interparticle distance was 10 nm.


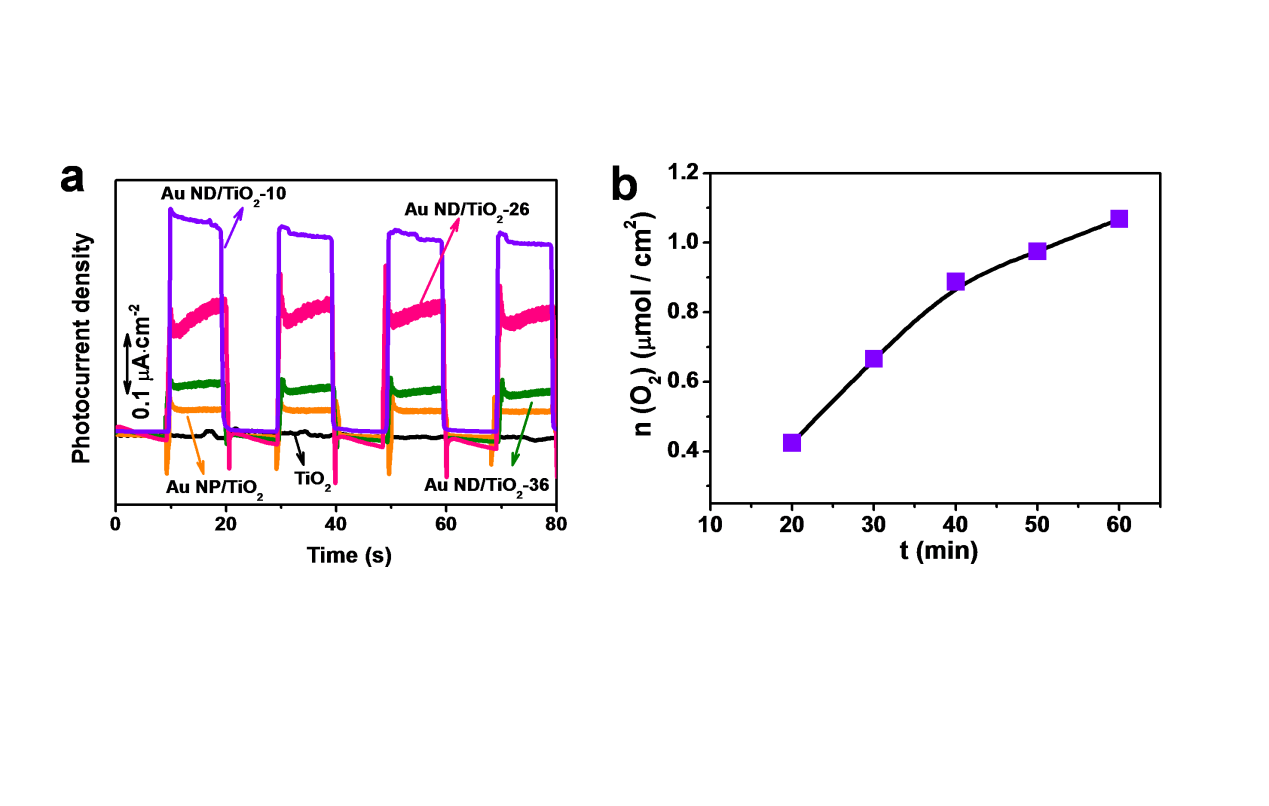


**Supplementary Figure 19.** (a) Chopped photocurrent–time curves of TiO_2_, Au NP/TiO_2_ and Au ND/TiO_2_ photoanodes. The current response is measured in 1 M Na_2_SO_4_ solution at 0.5 V versus Ag/AgCl under visible light. (b) Measured O_2_ in PEC system with mediated Au NDs/TiO_2_ at an applied voltage 0.5 V_Ag/AgCl_ under visible light irradiation.


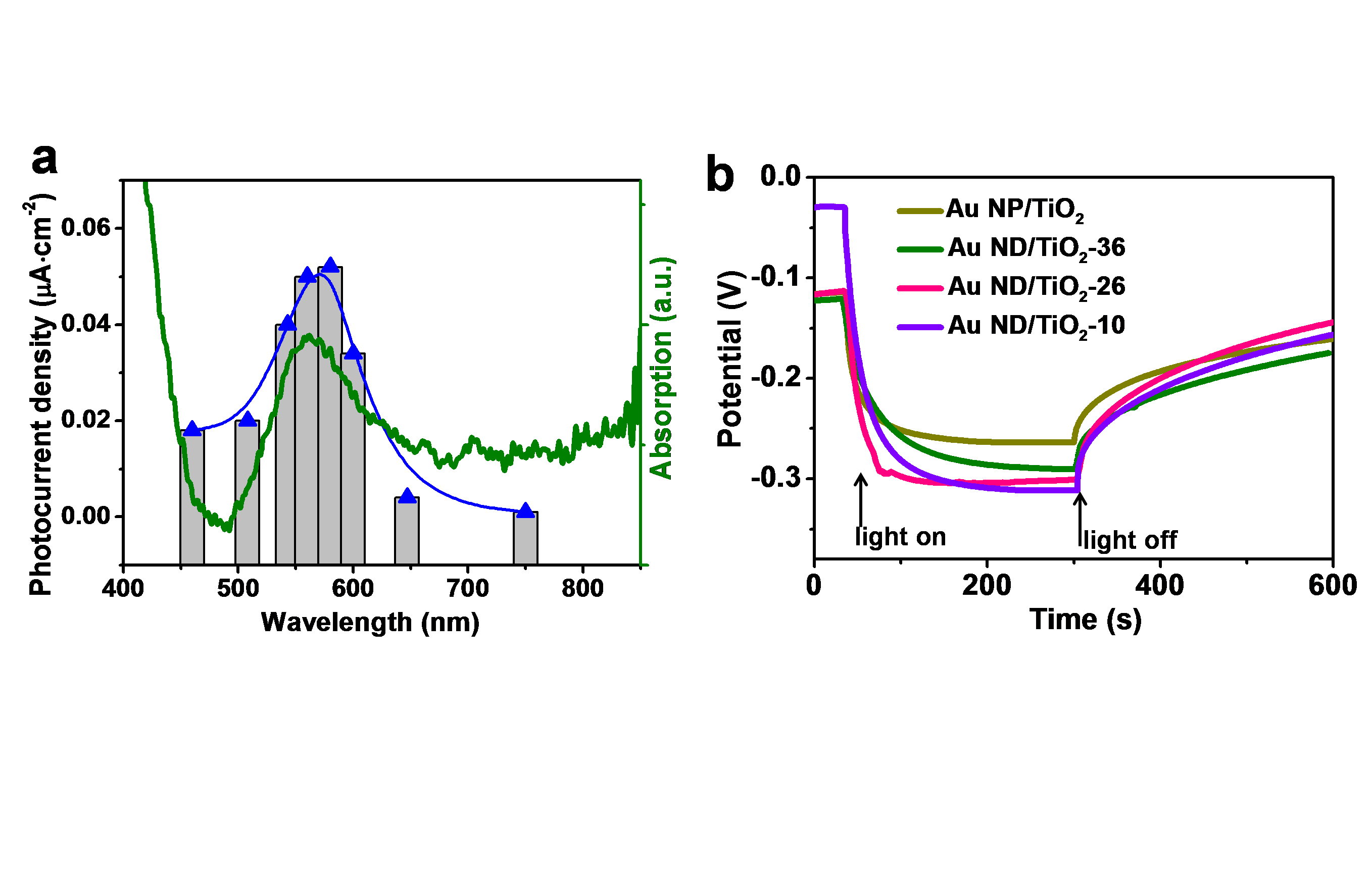


**Supplementary Figure 20.** (a) Action spectrum and absorption spectrum of Au ND/TiO_2_-10 nm in plasmonic water oxidation. (b) Open circuit potential (OCP) of Au NP/TiO_2_, Au ND/TiO_2_-36 nm, Au ND/TiO_2_-26 nm and Au ND/TiO_2_-10 nm under visible-light (>420 nm) illumination in 1 M Na_2_SO_4_ solution.


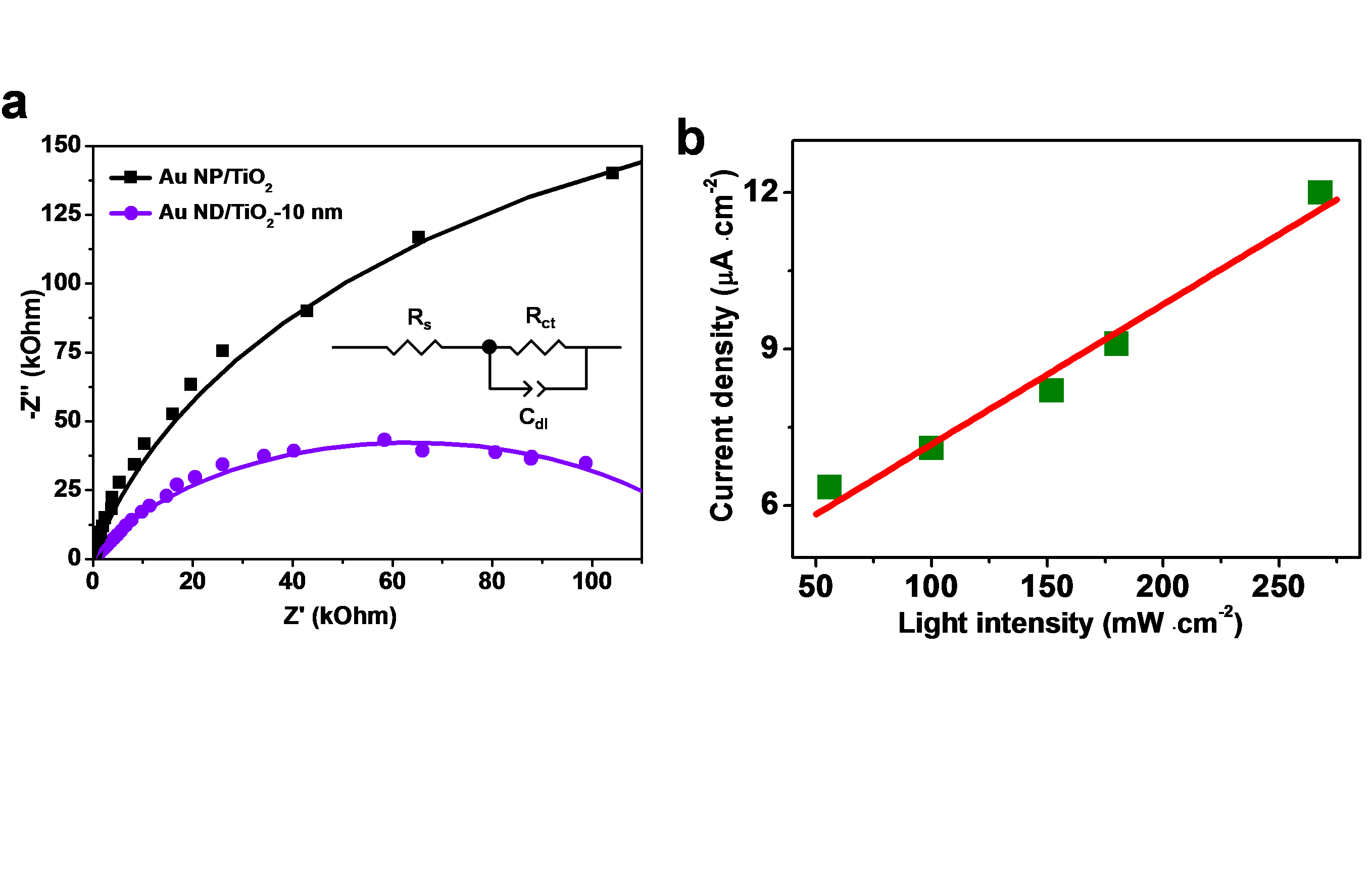


**Supplementary Figure 21.** (a) Tested (dot) and simulated (line) electrochemical impedance spectroscopy (EIS). Nyquist plots versus frequency of Au NP/TiO_2_ and Au ND/TiO_2_-10 nm photoanodes at 1.0 V in 1.0 M Na_2_SO_4_ under AM1.5G (1 sun) illumination at 100 mW cm^−2^. (b) Photocurrent density as a function of visible-light intensity (>420 nm) for Au ND/TiO_2_ sample. Photocurrent density exhibits a linear dependence on the light intensity.

**Supplementary Table S1.** Equivalent circuit fitting parameters of Au NP/TiO_2_ and Au ND/TiO_2_-10 nm photoanodes.


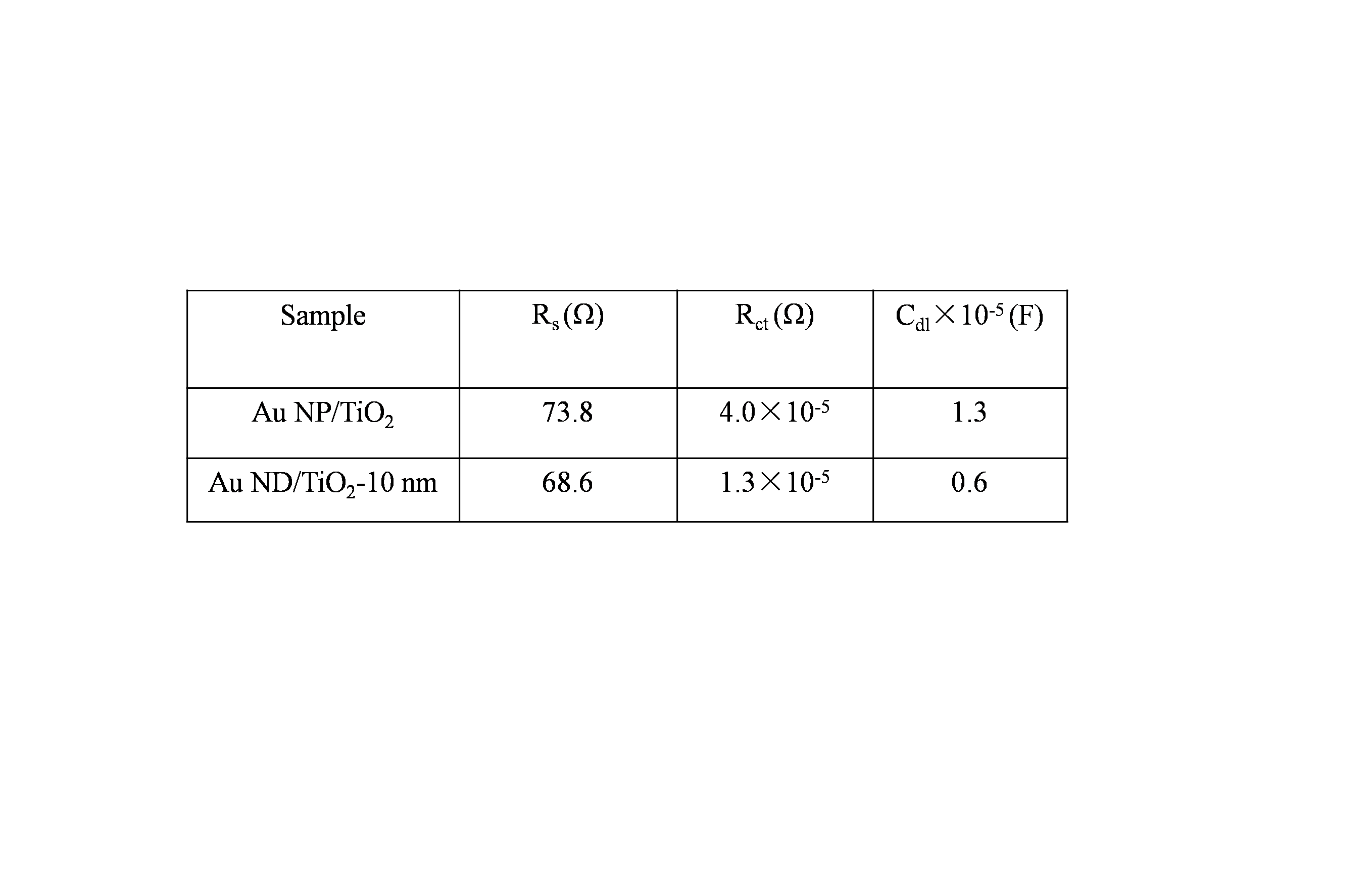


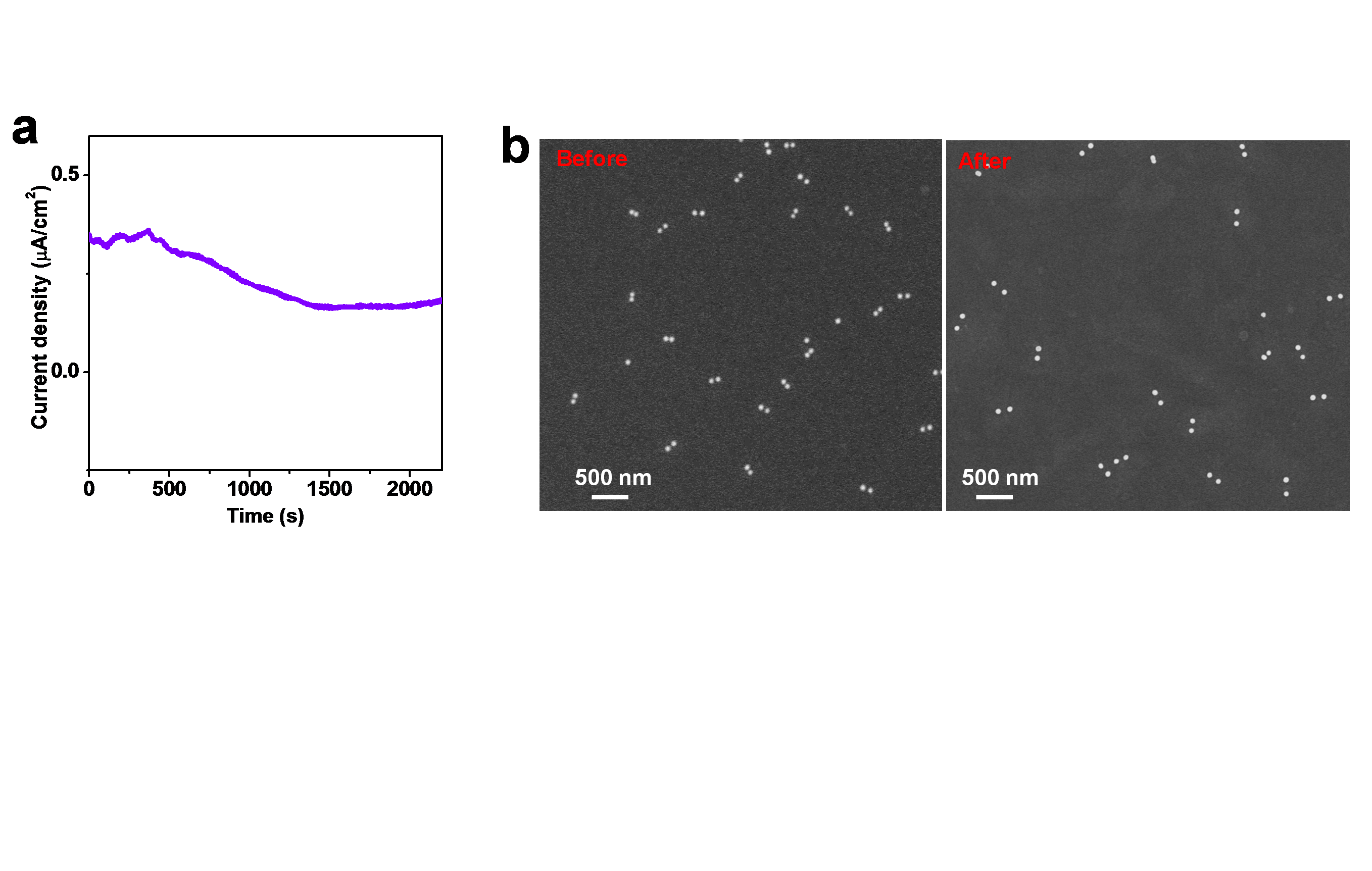


**Supplementary Figure 22.** (a) Photocurrent stability test of Au dimers/TiO_2_ sample at 0.25 V vs. Ag/AgCl under visible light illumination. (b) SEM images of Au dimers/TiO_2_ sample before and after photoelectrocatalytic reaction.

**References**

1. Fang WN, Jia SS and Chao J *et al.* Quantizing single-molecule surface-enhanced Raman scattering with DNA origami metamolecules. *Sci Adv* 2019; **5:** 4506.

2. Zhang YN, Chao J and Liu HJ *et al.* Transfer of Two-Dimensional Oligonucleotide Patterns onto Stereocontrolled Plasmonic Nanostructures through DNA-Origami-Based Nanoimprinting Lithography. *Angew Chem Int Ed* 2016; **55:** 8036-8040.

3. Bellot G, McClintock MA and Lin CX *et al.* Recovery of intact DNA nanostructures after agarose gel-based separation. *Nat Methods* 2011; **8:** 192-194.

4. Melitz W, Shen J and Kummel AC *et al.* Kelvin probe force microscopy and its application. *Surf Sci Rep* 2011; **66:** 1-27.

5. Robatjazi H, Zhao HQ and Swearer DF *et al.* Plasmon-induced selective carbon dioxide conversion on earth-abundant aluminum-cuprous oxide antenna-reactor nanoparticles. *Nat Commun* 2017; **8:** 27.

6. Johnson PB and Christy RW. Optical Constants of Noble Metals. *Phy Rev B* 1972; **6:** 4370-4379.

7. Kazuma E and Tatsuma T. In Situ Nanoimaging of Photoinduced Charge Separation at the Plasmonic Au Nanoparticle-TiO_2_ Interface. *Adv Mater Interfaces* 2014; **1:** 1400066.

8. Chen Q, Mao L and Li Y *et al.* Quantitative operando visualization of the energy band depth profile in solar cells. *Nat Commun.* 2015; **6:** 7745.

9. Strassburg E, Boag A and Rosenwaks Y. Reconstruction of electrostatic force microscopy images. *Rev Sci Instrum* 2005; **76:** 083705.

10. Yang JH, Guo YZ and Jiang RB *et al.* High-Efficiency "Working-in-Tandem" Nitrogen Photofixation Achieved by Assembling Plasmonic Gold Nanocrystals on Ultrathin Titania Nanosheets. *J. Am. Chem. Soc.* 2018; **140:** 8497-8508.

11. Jiang JJ, Huang ZQ and Xiang CX *et al.* Nanoelectrical and Nanoelectrochemical Imaging of Pt/p-Si and Pt/p(+)-Si Electrodes. *ChemSusChem* 2017; **10:** 4657-4663.

12. Kurnia F, Cheung J and Cheng X, *et al.* Nanoscale Probing of Elastic-Electronic Response to Vacancy Motion in NiO Nanocrystals. *ACS Nano* 2017; **11:** 8387-8394.

13. Govorov AO, Zhang H and Demir HV *et al.* Photogeneration of hot plasmonic electrons with metal nanocrystals: Quantum description and potential applications. *Nano Today* 2014; **9:** 85-101.

14. Harutyunyan H, Martinson AB and Rosenmann D *et al.* Anomalous ultrafast dynamics of hot plasmonic electrons in nanostructures with hot spots. *Nat.Nanotech.* 2015; **10:** 770-774.

15. Christopher P and Moskovits M. Hot Charge Carrier Transmission from Plasmonic Nanostructures. *Ann. Rev. Phy. Chem.,* 2017; **68:** 379-398.

16. Kumar MK, Krishnamoorthy S and Tan LK *et al.* Field Effects in Plasmonic Photocatalyst by Precise SiO_2_ Thickness Control Using Atomic Layer Deposition. *ACS Catal.* 2011; **1:** 300-308.

17. Kronik L and Shapira Y. Surface photovoltage spectroscopy of semiconductor structures: at the crossroads of physics, chemistry and electrical engineering. *Surf. Interface Anal.* 2001; **31:** 954-965.

18. Govorov AO, Zhang H and Gun'ko, YK. Theory of Photoinjection of Hot Plasmonic Carriers from Metal Nanostructures into Semiconductors and Surface Molecules. *J. Phy. Chem. C* 2013; **117:** 16616-16631.

19. Li YR, Guo Y and Long R *et al.* Steering plasmonic hot electrons to realize enhanced full-spectrum photocatalytic hydrogen evolution. *Chinese J. Catal.* 2018; **39:** 453-462.

20. Moskovits, M. The case for plasmon-derived hot carrier devices. *Nat. Nanotech.* 2015; **10:** 6-8.

21. Li GY, Mao B and Lan F *et al.* Practical aspects of single-pass scan Kelvin probe force microscopy. *Rev. Sci. Instrum.* 2012; **83:** 113701-1.

22. Zerweck U, Loppacher C and Otto T *et al.* Accuracy and resolution limits of Kelvin probe force microscopy. *Physical Review B* 2005; **71:** 125424-125429.

23. Fuchs F, Caffy F and Demadrille R *et al.* High-Resolution Kelvin Probe Force Microscopy Imaging of Interface Dipoles and Photogenerated Charges in Organic Donor-Acceptor Photovoltaic Blends. *ACS Nano* 2016; **10:** 739-746.

24. Lee SH, Lee SW and Oh T *et al.* Direct Observation of Plasmon-Induced Interfacial Charge Separation in Metal/Semiconductor Hybrid Nanostructures by Measuring Surface Potentials. *Nano Lett.* 2018; **18:** 109-116.

25. Junno T, Deppert K and Montelius L *et al.* Controlled Manipulation of Nanoparticles with an Atomic-Force Microscope. *Appl. Phys. Lett*. 1995; **66:** 3627-3629.

26. Galbrait.Lk and Fischer TE. Temperature-Dependence and Illumination-Dependence of Work Function of Gallium-Arsenide. *Suf. Sci.* 1972; **30:** 185-206.

27. Sheldon MT, van de Groep J and Brown AM *et al.* Plasmoelectric potentials in metal nanostructures. *Science* 2014; **346:** 828-831.

28. Qin ZP and Bischof JC. Thermophysical and biological responses of gold nanoparticle laser heating. *Chem. Soc. Rev.* 2012; **41:** 1191-1217.

29. Kronik L and Shapira Y. Surface photovoltage phenomena: theory, experiment, and applications. *Surf. Sci. Rep.* 1999; **37:** 1-206.

30. Gwo S, Chen HY and Lin MH *et al.* Nanomanipulation and controlled self-assembly of metal nanoparticles and nanocrystals for plasmonics. *Chem. Soc. Rev.* 2016; **45:** 5672-5716.

31. Zhang YJ, Pluchery O and Caillard L *et al.* Sensing the Charge State of Single Gold Nanoparticles via Work Function Measurements. *Nano Lett.* 2015; **15:** 51-55.

32. Dou MF and Persson C. Comparative study of rutile and anatase SnO_2_ and TiO_2_: Band-edge structures, dielectric functions, and polaron effects. *J. Appl. Phys.* 2013; **113.**
